# “*Sonochemically synthesized Ag(I) and Ni(II) schiff base complexes as efficient visible-light photocatalysts for dye degradation with DFT insights.”*

**DOI:** 10.1038/s41598-026-37498-8

**Published:** 2026-02-18

**Authors:** Aml M. Saleh, Amal G. Mahdy, Asmaa A. Hamed

**Affiliations:** Chemistry Department, Faculty of Science (Girls), Al-Azhar University, Nasr City, Cairo, Egypt

**Keywords:** Schiff base, Metal complexes, DFT, Photocatalytic degradation, Green synthesis, Sonication, Chemistry, Environmental sciences, Materials science, Nanoscience and technology

## Abstract

Water pollution caused by synthetic dyes is a pressing environmental concern, necessitating the development of highly efficient and reusable photocatalysts. Herein, we report the sonochemical synthesis and comprehensive characterization of nanoscale Ag(I) and Ni(II) Schiff base complexes derived from an isatin–sulfathiazole ligand (H₂L). The complexes were characterized by elemental analysis, molar conductance, magnetic susceptibility, FT-IR spectroscopy, ¹H NMR spectroscopy, UV–visible spectroscopy, XRD, TGA, and mass spectrometry. The obtained results confirmed their nanoscale dimensions, non-electrolytic behavior, and proposed tetrahedral geometries. TGA further assessed thermal stability and the presence of coordinated and lattice water molecules, with thermodynamic parameters calculated via the Coats–Redfern method. Complementary density functional theory (DFT) calculations provided detailed electronic and structural insights, supporting the proposed geometries and revealing reduced HOMO–LUMO energy gaps, distinct charge distribution patterns that highlight electron-rich and electron-deficient regions, and enhanced thermodynamic stability. Optical studies further confirmed the semiconducting nature of the synthesized compounds, consistent with their observed photocatalytic behavior. Both complexes exhibited remarkable photocatalytic performance under visible light, achieving Methylene Blue (MB) degradation efficiencies of 95.3% for the Ag(I) complex and 91.7% for the Ni(II) complex under optimized conditions (pH 11, 30 mg catalyst per 100 mL, and 10 ppm MB). Kinetic studies confirmed pseudo-first-order behavior, while recyclability tests demonstrated excellent stability and reusability over four consecutive cycles. These results highlight the potential of sonochemically synthesized Schiff base complexes as efficient, durable, and eco-friendly photocatalysts for wastewater treatment applications.

**Supplementary Information:**

The online version contains supplementary material available at 10.1038/s41598-026-37498-8.

## Introduction

Numerous toxins have been released into aquatic ecosystems as a result of the rapid development of industry and agriculture, endangering a variety of life forms^1^. This environmental problem is exacerbated by the fact that many textile industries use hazardous dyes in their dyeing procedures. An estimated 20% of global dye production is wasted, which has a detrimental effect on living organisms by reducing dissolved oxygen levels and limiting solar radiation penetration in aquatic environments. The majority of dyes are linked to serious conditions like genetic abnormalities and other fatal disorders^2^. As a result, eliminating these dyes from wastewater has become a top priority for scientists everywhere^3^. Activated carbon, chlorination, reverse osmosis, ultrafiltration, biodegradation, chemical oxygen demand (COD) reduction, and photodegradation are some of the methods that have been used in recent years to remove dangerous dyes from pollution^4^. Because photocatalysts may be recycled and used again, photocatalysis is the most cost-effective technique. Materials with photocatalytic qualities can produce electrons and holes in response to light, resulting in the development of promising photocatalysis components. Using photocatalyst nanoparticles, heterogeneous photocatalysis is a successful technique for getting rid of organic contaminants^5^. The photocatalysis has several advantages: (1) it breaks down pollutants without the use of auxiliary chemicals like ozone; (2) it works in ambient conditions; and (3) it uses mineralization to change dangerous organic pollutants into innocuous inorganic carbon dioxide and water^6^. On the other hand, Schiff bases, which are characterized by the imine (–C = N–) functional group, have garnered significant attention in coordination chemistry due to their ability to form stable complexes with a wide range of metal ions^7^. Schiff bases are used in a variety of ways because of their exceptional reactivity and traits, which are more effective when combined with transition metals^8^. Schiff base metal complexes have a wide range of uses in materials science, medicine, and catalysis, which makes them crucial for both basic and practical research^9–12^. In addition to their conventional uses, Schiff base metal complexes have recently drawn interest in environmental uses, especially in water treatment. Interestingly, because of their special qualities and possible uses, silver and nickel Schiff base complexes have been thoroughly investigated^13^. Sulfathiazole and isatin derivatives are known to have a significant, novel, and distinctive use in several chemistry-related fields^14,15^. Interestingly, the high-tech applications and important properties of complexes based on Ag(I) and Ni(II) have prompted much research. They have turned out to be promising possibilities because of their various physical and chemical features, such as redox, magnetic, catalytic, optical, and electronic effectiveness^16,17^. Motivated by the physicochemical and structural characteristics of Schiff base metal coordination compounds, we have created and described complexes of nickel and silver of a new Schiff base that was derived from isatin and sulfathiazole by an eco-friendly sonochemical approach. FT-IR, ¹H NMR, UV–visible, elemental analysis, magnetic moment, TGA, and X-ray diffraction (XRD) were among the spectroscopic methods used to examine their structural features. Building on this characterization, a detailed investigation of the photocatalytic activity of the synthesized compounds in Methylene Blue (MB) dye degradation is presented. To assess their efficiency under different conditions. We examined key factors influencing the degradation process, including photocatalyst dosage (20, 30, and 40 mg/100 mL dye solution), initial MB concentration (10, 20, and 30 ppm), and solution pH (3, 5, 7, 9, and 11). Additionally, the kinetics of MB photodegradation were analyzed using the first-order kinetic model, with rate constants calculated and compared.

## Experimental

### Chemicals

All chemicals, reagents, and solvents (isatin, sulfathiazole, NiCl_2_·6H_2_O, AgNO_3_, ethanol, diethyl ether, and glacial acetic acid) were of the highest available purity and analytical grade. They were obtained from Sigma-Aldrich and Merck (Egypt).

### Green synthesis of isatin-sulfa drug schiff base ligand (H₂L)

**Scheme 1:**
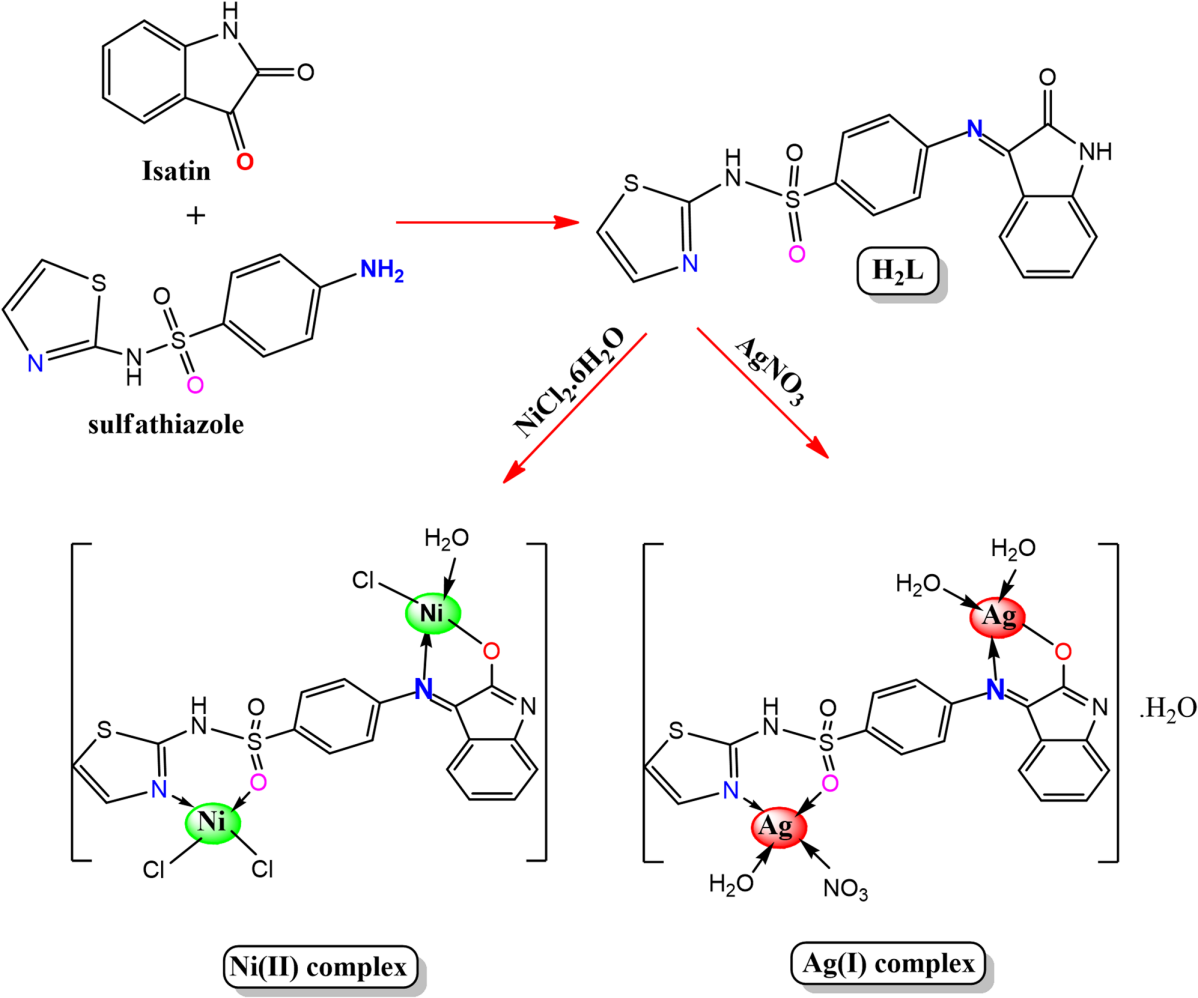
Synthesis pathway of the H₂L ligand and its Ag(I) and Ni(II) complexes.

The isatin-sulfa drug Schiff base ligand **(H₂L)** was prepared using a sonochemical method. An ethanolic solution (20 mL) of sulfathiazole (STHZ) (2.55 g, 1 mmol) was added to an ethanolic solution (20 mL) of isatin (IST) (1.47 g, 1 mmol) in a 1:1 molar ratio. A few drops of glacial acetic acid were added to the mixture. The mixture was sonicated for 3 h at 70 °C. The ethanolic solvent was evaporated at room temperature to get a red precipitate of the H₂L Schiff base ligand, which was washed with ethanol, followed by diethyl ether, and finally dried over anhydrous calcium chloride. The H₂L ligand was obtained as a red powder with an 87% yield (Scheme 1).

### Eco-friendly metal complex synthesis

Nanoscale Ag(I) and Ni(II) complexes were prepared by adding an aqueous solution of NiCl_2_·6H_2_O and AgNO_3_ salts (10 mmol) dropwise to an ethanolic solution of H₂L (5 mmol), 2:1 metal-to-ligand molar ratio. The resulting reaction mixture was sonicated for 2 h at 70 °C, during which a distinct color change and precipitate formation were observed. The precipitated complexes were filtered and repeatedly washed in ethanol and diethyl ether before being dried in a desiccator over anhydrous calcium chloride. Nanoscale Ag(I) complex was obtained as a dark brown powder with an 80% yield, while the nanoscale Ni(II) complex appeared as a faint brown powder with a 79% yield (Scheme 1).

### Instruments

An electrothermal melting point apparatus with an open capillary outlet was used to determine the melting points of the synthesized complexes. IR spectra contained in the range of 400–4000 cm^− 1^ were recorded by a Shimadzu infrared spectrophotometer via KBr discs. Using DMSO-d₆ as a solvent, signals in the ¹H NMR spectra were detected using a Bruker 400 MHz spectrometer (chemical shifts (δ) expressed in parts per million (ppm)). A Perkin-Elmer UV–visible spectrophotometer was utilized to capture the UV/visible absorption spectra of the prepared compounds. The JEN conductometer device recorded specific conductance results at room temperature (values expressed in Ω^−1^ cm^2^ mol^− 1^). A Gouy magnetic balance was used to assess magnetic sensitivity. A Perkin-Elmer CHN analysis equipment was used for performing elemental analyses at Cairo University’s Micro Analytical Unit in Cairo, Egypt. The mass spectra of the synthesized complexes were determined using the EI-Mass GC-2010 Shimadzu instrument. TGA measurements were carried out on a DTG-50 Shimadzu (45–800 °C) under a nitrogen atmosphere, with a heating rate of 10 °C/min. XRD patterns were recorded using a Philips diffractometer with a monochromatized CuKα radiation source. Mass spectra were obtained using a Finnigan MAT SSQ7000 spectrometer with electron ionization (EI) at 70 eV.

### DFT methodology

All theoretical calculations were performed using Gaussian 09, based on density functional theory (DFT)^18^. The Gauss View 6.0.16 program was used to create geometric structures^19^. The 6-31G(d, p) basis set was adopted for C, H, N, O, and S atoms, while the LanL2DZ basis set was adopted for Ag and Ni metal ions^10^. The electronic chemical descriptors, such as frontier orbitals (HOMO and LUMO), energy gap (ΔE), chemical hardness, electronic chemical potential, electrophilicity index, dipole moment, and atomic Mulliken charges, were calculated.

### Calculating the charge zero point

The point of zero charge (pHpzc) for the prepared Ag(I) and Ni(II) complexes was determined following the reported procedure^6^. In brief, 50 mL of 0.01 M KNO₃ solution was prepared in separate vessels covering a pH range of 3.0–10.0, adjusted using 0.1 M HCl and 0.1 M NaOH. After stabilization, 0.015 g of each complex was added to the solutions, shaken for 2 h, and allowed to stand for 2 days. After this period, the solutions were filtered, and the final pH was measured.

### Photocatalytic procedure for dye degradation

In a cylinder-shaped Pyrex reactor, 100 mL of Methylene Blue (MB) dye solutions at different concentrations (10, 20, and 30 ppm) were exposed to visible light to evaluate the photocatalytic effectiveness of the generated complexes. To provide visible light, a 60-watt tungsten lamp with a wavelength of λ > 420 nm was placed above the reactor. The impact of the catalyst quantity was evaluated using catalysts of varying weights (20, 30, and 40 mg/100 mL of the dye solution). To achieve adsorption-desorption equilibrium, the solutions were agitated in the dark for 30 min before radiation treatment. Throughout the degrading process, samples were continuously centrifuged to separate the solid particles for further examination. The concentrations of the Methylene Blue (MB) Dye solution were measured using a PerkinElmer Lambda-365 ultraviolet-visible spectrophotometer with a peak absorption wavelength of 664 nm. Once the photocatalyst and reactant solution had been separated using a centrifuge, we conducted further testing. By employing 0.1 mol/L solutions of HCl and NaOH, respectively, we examined the process by which the (MB) solution’s initial pH was altered to 3, 5, 7, 9, and 11 to study the effect of solution pH. For every case, the photodegradation (%) of MB was calculated under the same experimental setup. The photodegradation of MB dye without a catalyst was studied, and no significant degradation was observed, with an efficiency of approximately 1.5%. Equation (1) was used to determine the percentage of dye degradation^20^:

1 $$\:\mathrm{d}\mathrm{e}\mathrm{g}\mathrm{r}\mathrm{a}\mathrm{d}\mathrm{a}\mathrm{t}\mathrm{i}\mathrm{o}\mathrm{n}\:\mathrm{\%}=\left(\right({A}_{o}-A)/{A}_{o})\times\:100$$

where *A*_*o*_= initial MB dye absorbance, and *A*_*t*_= absorbance of the MB dye solution after the degradation time ‘t’.

## Results and discussion

### Elemental analysis

In the experimental section, the prepared complexes were subjected to physical property characterization, including the determination of their melting points, colors, and percentage yields. The melting points of all complexes were found to exceed 300 °C, and they all exhibited distinct colors. The proposed chemical structure of the complexes agreed with the elemental studies (C, H, and N). The investigations verified that the Schiff base ligand (H₂L) exhibited a 1:2 (L: M) molar ratio upon complexation with the metal ions. According to this observation, each metal ion is coordinated to a single H₂L molecule, forming stable complexes with well-defined stoichiometry. Our comprehension of the Schiff base complexes’ chemical properties was improved, and their thorough characterization was made easier by the valuable information provided by the physical and elemental investigations of their stoichiometry, composition, and structural features^21^. All the data is shown in Table 1.

### Molar conductivity measurements

The molar conductance values of the synthesized metal complexes were measured at room temperature in DMF (1 × 10⁻³ M) to evaluate their degree of dissociation in solution and ionic nature. The findings demonstrated the non-electrolytic character of all the complexes by showing molar conductivity readings less than 50 Ω^−1^ cm^2^ mol^− 1^.

**Table 1:** Data from physical-analytical and elemental analyses of H₂L ligand and its metal complexes.

Compounds M. F.	M. Wt	Color Yield (%)	M. *P*. (ºC)	Found/(Calc.) %			Λ Ω^−1^ cm^2^ mol^− 1^
				C	H	*N*	
H₂L C_17_H_12_N_4_O_3_S_2_	384.43	Dark red 87		53.11 (53.32)	3.15 (3.20)	14.57 (14.59)	-
[Ag_2_(HL)(NO_3_)(H_2_O)_3_]·H_2_O C_17_H_19_N_5_O_10_S_2_Ag_2_	715.21	Dark brown 80	> 300	28.55 (28.70)	2.40 (2.56)	9.79 (9.88)	10.5
[Ni_2_(HL)(Cl)_3_(H_2_O)] C_17_H_13_Cl_3_N_4_O_4_S_2_Ni_2_	625.19	Faint brown 79	> 300	32.66 (32.40)	2.10 (2.26)	8.96 (8.52)	15

### IR spectral studies

**Fig. 1:**
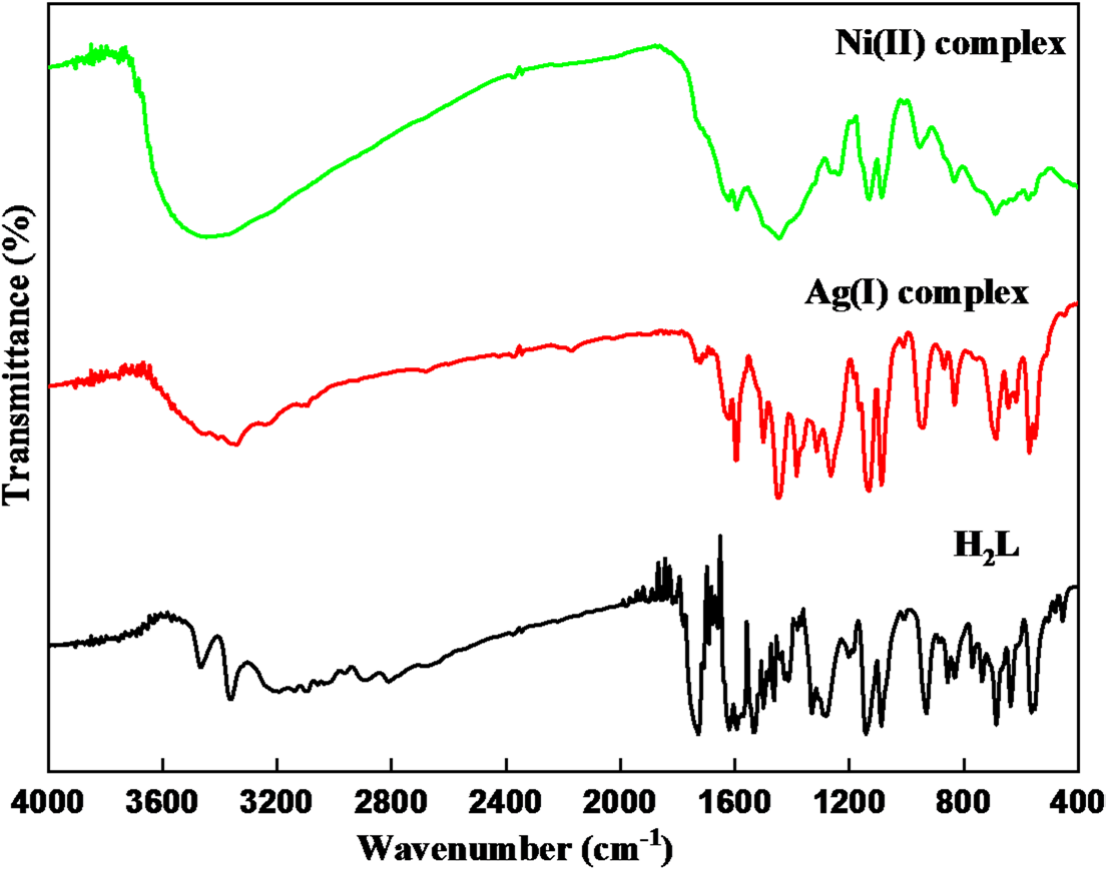
FT-IR spectra of the H₂L ligand and its Ag(I) and Ni(II) complexes.

FT-IR spectra of H₂L and its metal complexes were compared to ensure H₂L coordination to the metal ions. The spectra are shown in Fig. 1, and the assignments of selected bands are presented in Table 2. The absence of characteristic bands corresponding to the amino group of sulfathiazole and the carbonyl group of isatin, along with the appearance of a new intense band at 1593 cm^-^¹, confirms the formation of the azomethine group (C = N) in H₂L^22,23^. The FT-IR spectrum of H₂L shows absorption bands at 3468 and 3364 cm⁻¹, which are assigned to the sulfonamide ν(N-H) and isatin units ν(N-H), respectively. Additional bands at 1728, 1462, and 1141 cm⁻¹ correspond to ν(C = O), ν(SO₂)_sym_, and ν(SO₂)_asym_, respectively^24,25^.

Here is a comparison of the FT-IR spectra of metal complexes and H₂L:

- The broad bands in the 3600–3000 and 3700–2500 cm⁻¹ ranges in the metal complexes may arise from the presence of water ν(OH), as confirmed by the elemental and thermal analyses^26,27^. The coordinated water molecules typically exhibit a characteristic peak at 833 cm⁻¹, corresponding to the bending mode of the H₂O molecule^21^.
- NH, C = O, and/or OH bands are absent in the complexes due to deprotonation of the enolic proton, which occurs as a result of tautomerism in solution^28^.
- The coordination of azomethine nitrogen to the metal ions is indicated by the strong bands at 1597 and 1595 cm⁻¹ that are caused by the shifting of azomethine to higher wave numbers in metal complexes^11,29^.
- The vibrational frequencies due to the SO_2_ group are shifted to higher frequencies, reflecting the participation of the sulfonamide nitrogen in chelation^30^.
- The Ag(I) complex exhibits strong bands at 1384 and 867 cm⁻¹, corresponding to the nitrate group in a unidentate coordination mode^31^.
- Additional evidence of the bonding is provided by the discovery of new bands in the low-frequency region of metal complexes’ FT-IR spectra at 644 and 690 and 570 and 574 cm⁻¹, which are caused by ν(M-O) and ν(M-N)^30,32^.

**Table 2:** H₂L ligand and its Ag(I) and Ni(II) complexes infrared absorption frequencies (cm⁻¹).

Compounds	ⱱ(*N*-H)/H_2_O	ⱱ(C = *N*)_azo_	ⱱ(SO_2_)_sym_/_asym_	ⱱ(NO_3_^−^) Coord.	ⱱ(M-O)	ⱱ(M-*N*)
H_2_L	3468, 3364	1593	1462/1141	-	-	-
Ag(I) complex	3600 − 3000	1597	1446/1134	1384/867	644	570
Ni(II) complex	3700 − 2500	1595	1446/1130	-	690	574

### ¹HNMR spectra study

**Fig. 2:**
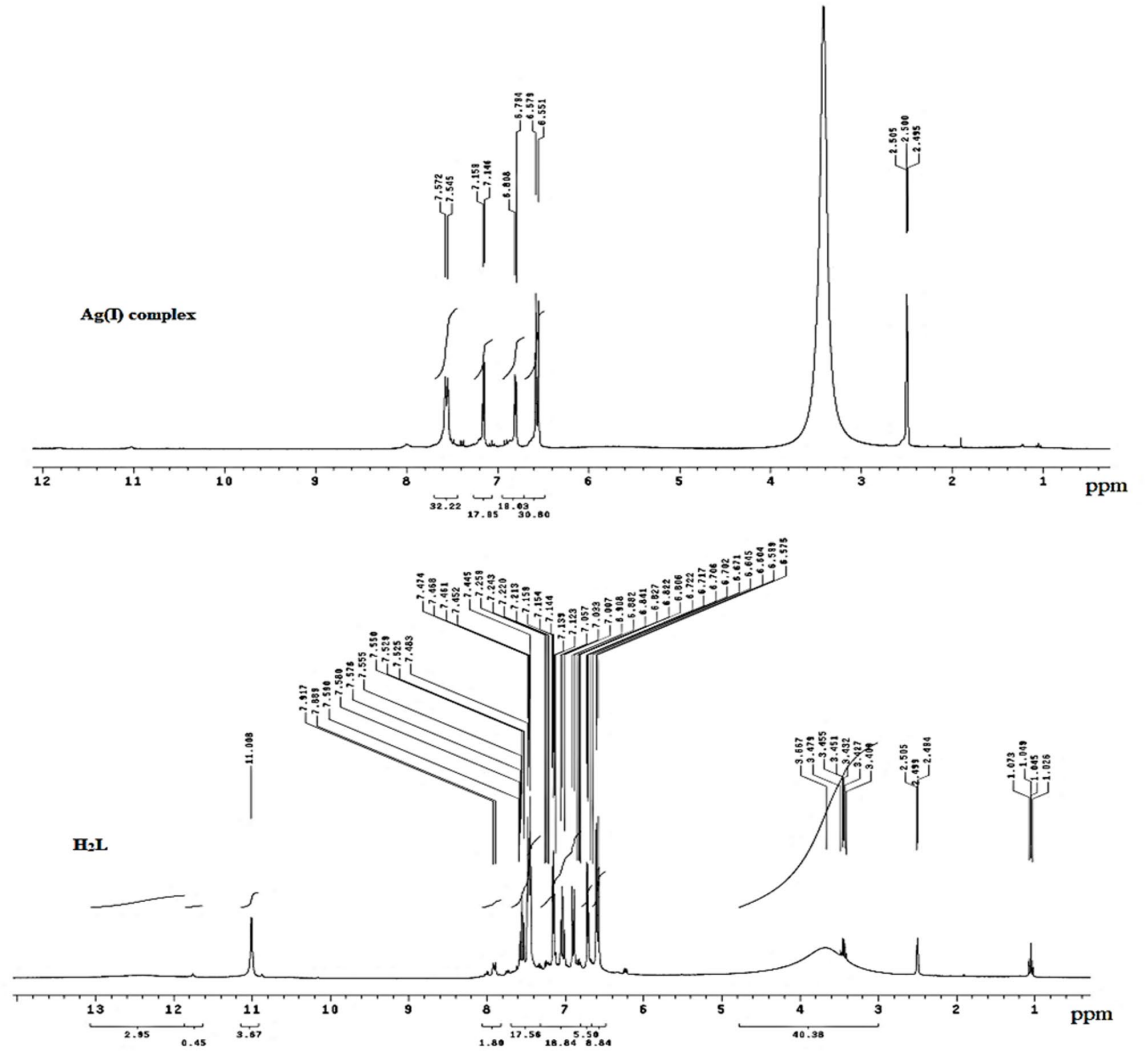
¹H NMR spectra of the H₂L ligand and its Ag(I) complex.

The variance of the ¹H NMR of the H₂L ligand and its Ag(I) complex is denoted in Table 3; Fig. 2. H₂L Schiff base ligand exhibited a distinct proton signal corresponding to NH-isatin at δ = 11.00 ppm^33,34^. In the Ag(I) complex, this signal is absent, suggesting that the NH-isatin group underwent tautomerization with the C = O-isatin group, resulting in the formation of a bond between the oxygen and the silver atom^28^. All signals due to aromatic/hetero protons were found to be multiples in their expected regions^24^. Proton signals due to thiazole ring-2 H showed shifting to 6.55–6.80 ppm in the Ag(I) complex due to coordination. Additionally, a broad signal at δ = 3.41 ppm in the Ag(I) complex can be attributed to water molecule protons, supporting the proposed complex formula^35^.

**Table 3:** ¹H NMR spectra of H₂L ligand and its Ag(I) complex.

Group signals	Assignments (δppm)	
	H_2_L	Ag(I) complex
(NH)-isatin	11.00	---
4 H-indole moiety	7.00–7.44.00.44	7.14–7.15
4CH-Ar (sulfonamide)	7.45–7.91	7.54–7.57
2 H-thiazole	6.57–6.90	6.55–6.80
H_2_O coordinated	---	3.41

### Electronic spectral study (UV–Visible)

**Fig. 3:**
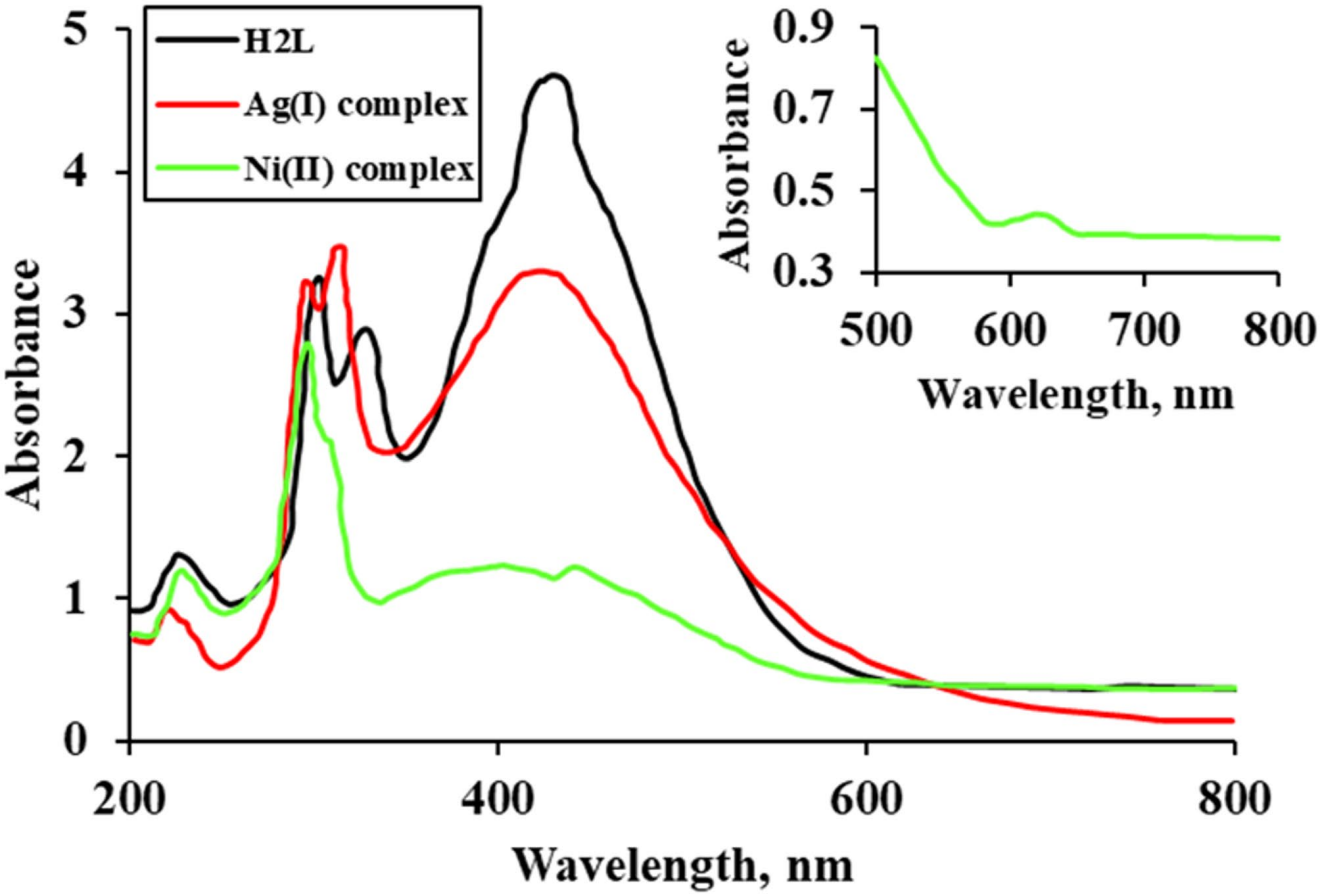
UV–Vis spectra of the H₂L ligand and its Ag(I) and Ni(II) complexes.

The UV–visible spectral study of the H₂L ligand and its Ag(I) and Ni(II) complexes was carried out in DMF at a concentration of 10⁻⁴ M over the 200–800 nm range at room temperature, as shown in Fig. 3. The spectrum of the H₂L ligand showed electronic absorption bands ranging from 296 to 420 nm, which are characteristic of intra-ligand transitions. The bands at 296 nm and 321 nm are attributed to the π-π* transition of the aromatic and azomethine chromophores. The band at 420 nm is assigned to the n-π* transition of the azomethine functional group^36^. These bands slightly shifted upon complex formation, demonstrating the coordination of the ligand to the metal ions. The electronic absorption bands in the Ag(I) and Ni(II) complexes arise from π→π*, n→π*, and ligand-to-metal charge transfer (LMCT) transitions and are observed in the ranges 298–423 nm and 298–440 nm, respectively^37–40^,. The Ag(I) complex exhibits a filled *d¹⁰* orbital configuration, which prevents significant d–d transitions, supporting a tetrahedral geometry. The Ni(II) complex displays a weak band at 626 nm, assigned to the ³T₁(F) → ³T₁(P) transition. The relatively low intensity of this band is consistent with the generally low molar absorptivity of d–d transitions compared to charge-transfer bands. Its magnetic moment of 4.84 B.M. further confirms a tetrahedral geometry^13^.

### Optical properties studies

**Fig. 4:**
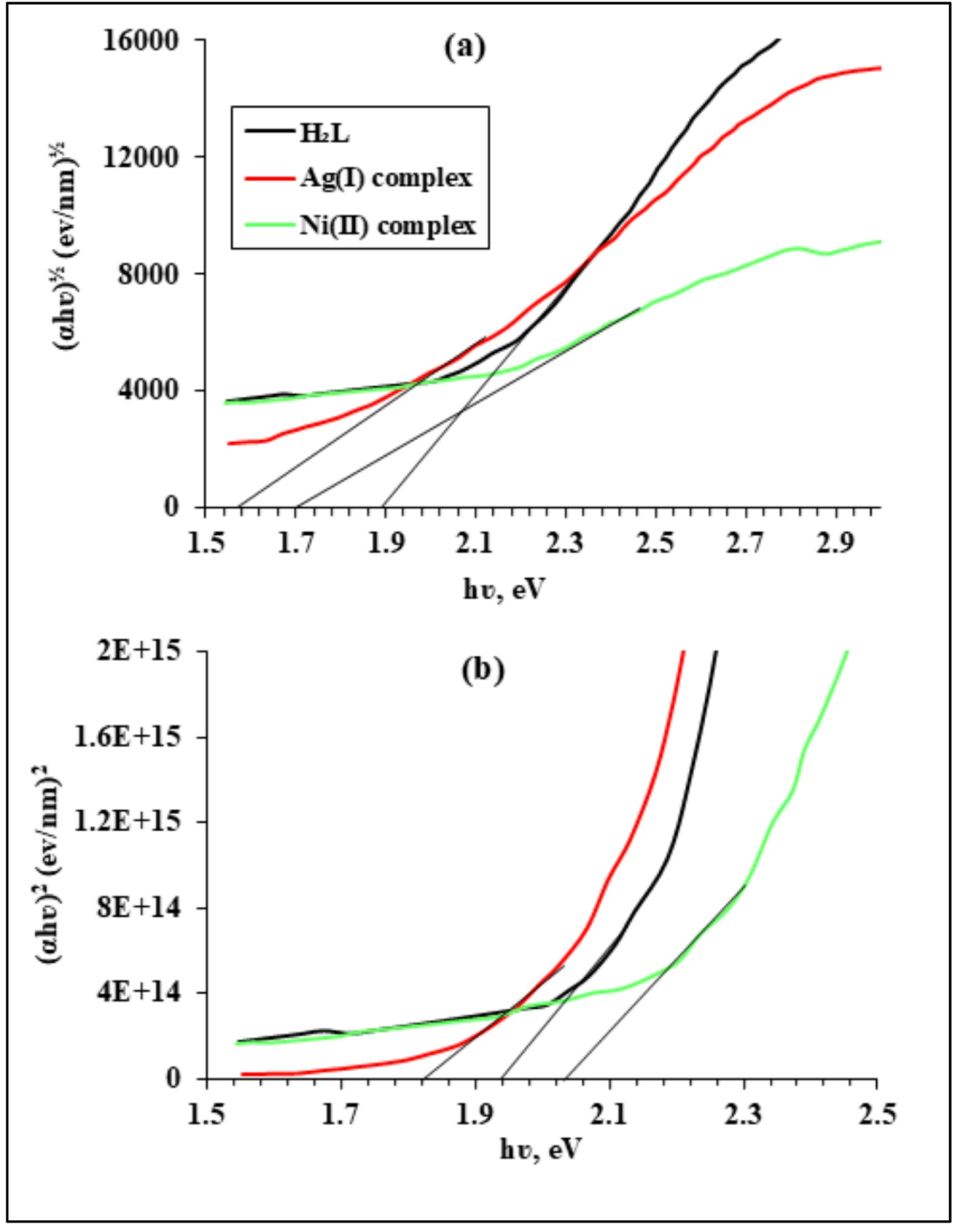
Tauc plots of the H₂L ligand and its Ag(I) and Ni(II) complexes obtained from UV–Vis absorption data: (**a**) plots of $\left( \alpha h \nu \right)^{1/2}$versus $\:h\nu\:$, corresponding to indirect transitions, and (**b**) plots of $\left( \alpha h \nu \right)^2$versus $\:h\nu\:$, corresponding to direct transitions. The optical band gap energy ($\:{E}_{g}$) values were estimated by linear extrapolation of the linear regions.

With the Tauc plot approach, the band gap energy of H₂L and its metal complexes was determined. This involved plotting (αhʋ)ⁿ against (hʋ) and finding the band gap values by extrapolating the linear part of the curve to the energy axis, as shown in Figs. 4a **and b.** The absorption coefficient α is determined by 2.303 A/d in the equation αhv = B(hν- E_g_)ⁿ, where A stands for absorbance and d for optical path length. For indirect permitted transitions, the constant n takes the value ½, while for direct permitted transitions, it takes the value 2. Additionally, B is the proportionality constant, E_g_ represents the band gap energy, and hν corresponds to the photon energy. According to the Tauc plots, the H₂L and its Ag(I) and Ni(II) complexes have optical band gap energies of 1.88, 1.58, and 1.7 eV for indirect permitted transitions, respectively. For directly allowed transitions, the optical band gap energies are 1.94, 1.82, and 2.02 eV for H_2_L and its Ag(I) and Ni(II) complexes, respectively. According to the measured E_g_ value, the produced compounds appear to have semiconductor properties^41–45^.

### Thermal analysis

The thermal behavior of H₂L and its metal complexes was investigated using thermogravimetric and differential thermogravimetric analysis (TGA/DTG) under a nitrogen atmosphere, with a gas flow rate of 20 mL min⁻¹, a heating rate of 10 °C min⁻¹, and a temperature range from room temperature to 800 °C. Thermal analysis was employed to assess the stability of the synthesized compounds and to determine whether water molecules are present as coordinated or lattice water within the complexes.

**Fig. 5:**
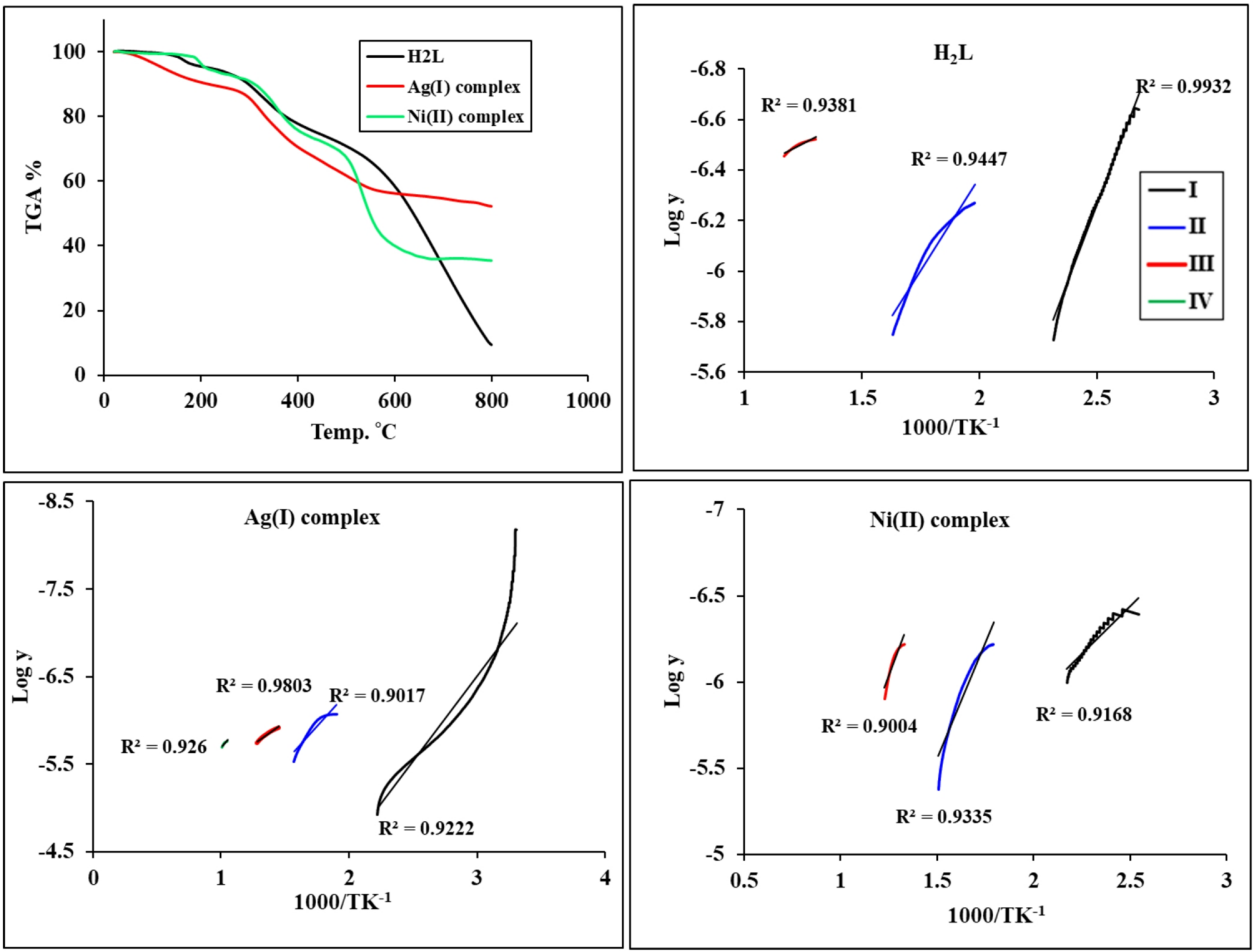
Linear fitting plots and thermogravimetric (TGA) curves of H₂L ligand and its Ag(I) and Ni(II) complexes.

Figure 5; Table 4 summarize the thermal analysis results, including the temperature ranges of decomposition, decomposition steps, and experimental and calculated weight-loss percentages. Compared with the free ligand H₂L, the metal complexes exhibit similar decomposition patterns, reflecting structural similarities and comparable thermal stability. The H₂L ligand undergoes three main decomposition stages. At a 100–180 °C temperature range, a 4.74% (calc. 4.43%) mass loss is equivalent to removing NH_3_. At temperatures between 232 and 376 °C, a mass loss of 20.34% (calc. 20.32%), corresponding to the removal of C₆H₆, represents the second stage of decomposition. The final step occurs within the range of temperatures (494–777 °C), a 62.86% (calc. 62.75%) loss of mass corresponding to taking out C_7_H_3_N_3_O_3_S_2_. The 87.94% (calc. 87.50%) total mass loss that remains 4 C as the last residue is 12.06% (calc. 12.49%).

The **[Ag**_**2**_**(HL)(NO**_**3**_**)(H**_**2**_**O)**_**3**_**]**·**H**_**2**_**O** complex underwent four decomposition stages. With a mass loss of 9.49% (calculated as 10.00%), the first stage takes place between 25 and 175 °C and is equal to the removal of 4H_2_O hydr., and Coord. A second, temperature-dependent breakdown stage of 250–376 °C produced a mass loss of 20.00% (calc. 20.29%), due to the rejection of SO_2_, HNO, 2NH_3_, and CH_4_. The third step at 417–548 °C with mass loss of 12.80% (calc. 13.15%) corresponds to the elimination of SO and NO_2_. The final step between 677 and 737 °C caused a loss of mass of 1.52% (calc. 2.00%) due to the absence of ½N_2_, with the residual residue consisting of 16 C, and 2Ag found 56.21% (calc. 57.03%).

The **[Ni**_**2**_**(HL)(Cl)**_**3**_**(H**_**2**_**O)] complex** decomposed in three steps. H_2_O Coord. and ½N_2,_ a 4.59% (calc 5.12%) mass loss was released during the first phase of breakdown. The second stage of decomposition, with 18.85% (calc. 19.25%) mass loss, corresponds to 1½Cl_2_, and ½N_2_. Weight loss in the last stage was 33.19% (calc. 32.51%), which matches the removal of C_6_H_5_S, 2NH_3_, SO, and C. The remaining residue was 43.37% (calculated 43.10%) of 10 C and 2NiO.

### Kinetic and thermodynamic parameters

Kinetic and thermodynamic parameters for H₂L and its complexes are calculated by the Coats–Redfern method under non-isothermal conditions, shown in Fig. 5^46^. The other parameters, such as S* (entropy of activation), H* (enthalpy of activation), and G* (free energy change of activation), can also be calculated using Eyring equations. The information gathered is displayed in Table 5: H* = E* - RT, S* = 2.303[log (Ah/KTs)]R, G* = H* − T_S_*. The Planck and Boltzmann constants are h and K^47^. Furthermore, positive E_a_ values ensured typical degradation processes. Moreover, positive “ΔG*” values verify that not all decomposition events are spontaneous, and that the final product’s free energy is greater than that of the starting component. Negative ΔS* values demonstrated that the activated complexes were more ordered than the reactants, while negative ΔH* values further suggested exothermic breakdown^48^.

**Table 4:** TGA decomposition stages, temperature ranges, and weight-loss percentages of the H₂L ligand and its metal complexes and their weight loss.

Compounds	Stages	Temp. Range, (°C)	DTG max (°C)	Mass loss (%)		Evolved moiety	Residue moiety
				Found	Calc.		
H_2_L	I	100–180	164	4.74	4.43	NH_3_	C_17_H_9_N_3_O_3_S_2_
	II	232–376	329	20.34	20.32	C_6_H_6_	C_11_H_3_N_3_O_3_S_2_
	III	494–777	675	62.86	62.75	C_7_H_3_N_3_O_3_S_2_	4 C
Ag(I) complex	I	25–175	127	9.49	10.00	4H_2_O _hydrated+ Coord_.	C_17_H_11_N_5_O_6_S_2_Ag_2_
	II	250–376	340	20.00	20.29	SO_2_, HNO, 2NH_3_, CH_4_	C_16_N_2_O_3_SAg_2_
	III	417–548	518	12.80	13.15	SO, NO_2_	C_16_NAg_2_
	IV	677–737	713	1.50	1.95	½N_2_	16 C_+_ 2Ag
Ni(II) complex	I	120–205	198	4.59	5.12	H_2_O Coord, ½N_2_	C_17_H_11_Cl_3_N_3_O_3_S_2_Ni_2_
	II	284–393	357	18.85	19.25	3Cl, ½N_2_	C_17_H_11_N_2_O_3_S_2_Ni_2_
	III	453–565	535	33.19	32.51	C_6_H_5_S, 2NH_3_, SO, C	10 C+ 2NiO

**Table 5:** Kinetic behavior of H₂L ligand and its complexes using the Coats–Redfern method under non-isothermal conditions.

Compounds	Steps	Decomposition Temp. ˚C	A (min^− 1^)	∆H^#^ (J/mol)	∆S ^#^ (J/mol.K)	∆G^#^ (J/mol)	Ea (J/mol)	*R* ^2^
H_2_L	I	100–180	648.319	−3580.222	−188.531	78619.161	47.298	0.99
	II	232–376	0.367	−5271.555	−259.786	160211.847	28.285	0.94
	III	494–777	1.6 × 10^− 4^	−5601.558	−324.55	213144.972	6.122	0.94
Ag(I) complex	I	25–175	261.363	−3290.773	−201.251	77209.646	37.227	0.92
	II	250–376	0.814	−5069.236	−252.831	149916.451	30.924	0.90
	III	417–548	0.023	−6811.381	−285.118	227270.539	19.339	0.98
	IV	677–737	0.068	−8174.186	−277.412	265353.861	29.334	0.92
Ni(II) complex	I	120–205	0.15	−3897.568	−264.722	120786.298	21.152	0.91
	II	284–393	57.468	−5181.326	−217.622	131703.155	51.954	0.93
	III	453–565	11.911	−6664.155	−232.802	181440.17	58.405	0.90

### Mass spectral study

**Fig. 6:**
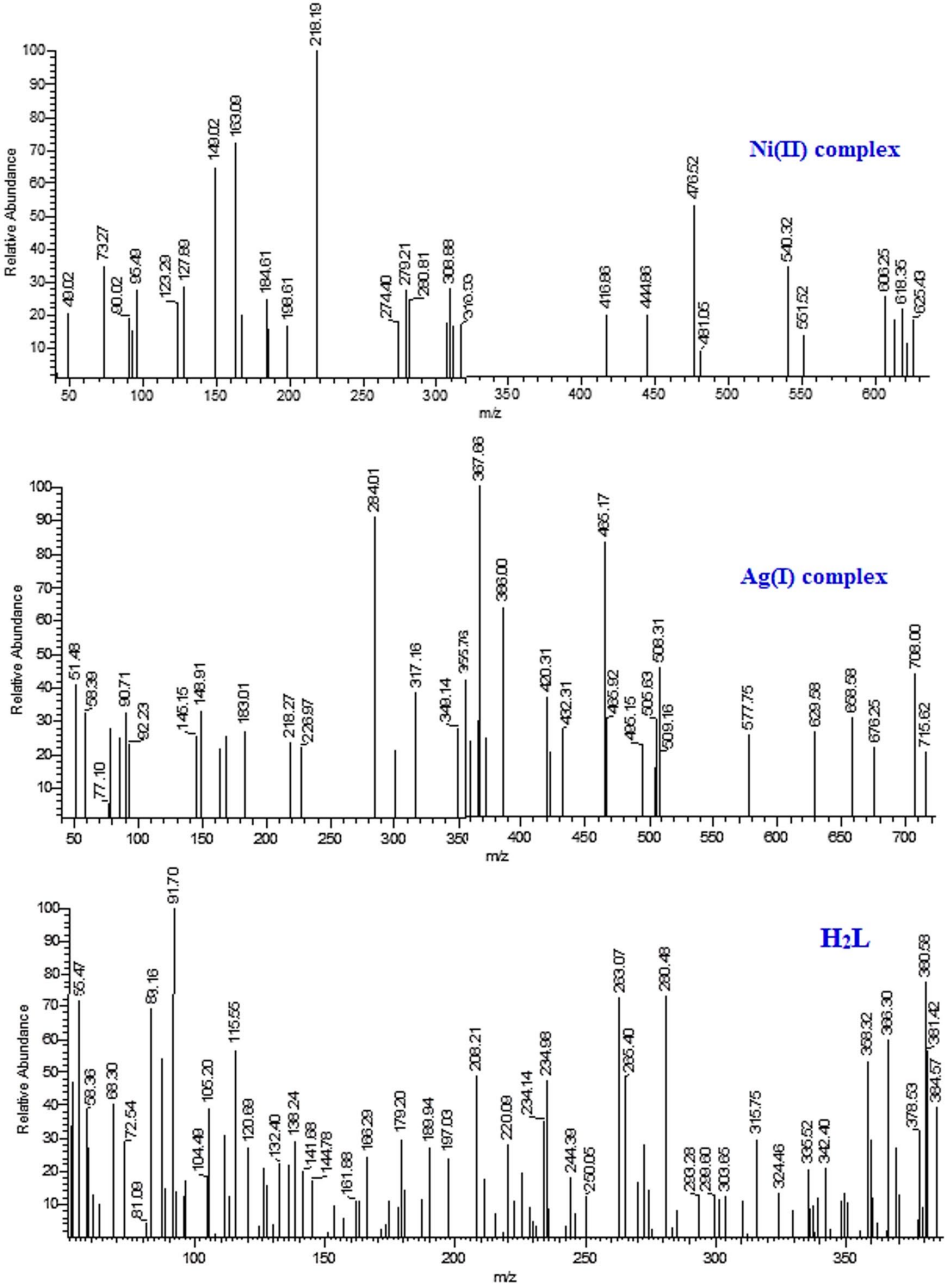
Mass spectra of H₂L ligand and its Ag(I) and Ni(II) complexes, showing molecular ion peaks and fragmentation patterns.

The molecular weights and fragmentation patterns of the H_2_L free ligand and its metal complexes were determined by analyzing their mass spectra (MS), as shown in Fig. 6. H_2_L free ligand presented a molecular ion peak at m/z = 384.57 (39.19%), which is in accordance with its molecular weight (molecular formula) of 384.43 (C_17_H_12_N_4_O_3_S_2_). For Ag(I) and Ni(II) complexes, the parent ion peaks at m/z = 715.62 (20.31%) and 625.43 (18.27%) are in good agreement with their molecular weights (molecular formula) of 715.21 [Ag_2_(HL)(NO_3_)(H_2_O)_3_]·H_2_O and 625.19 [Ni_2_(HL)(Cl)_3_(H_2_O)], respectively. The stability of the fragments is also indicated by the various peaks in the mass spectra of the H_2_L free ligand and its complexes, which show sequential degradation with significant intensity. Figs. S1–S3 show the suggested mass fragmentation pathway for the original ligand and its complexes.

### X-ray diffraction (XRD) analysis

**Fig. 7:**
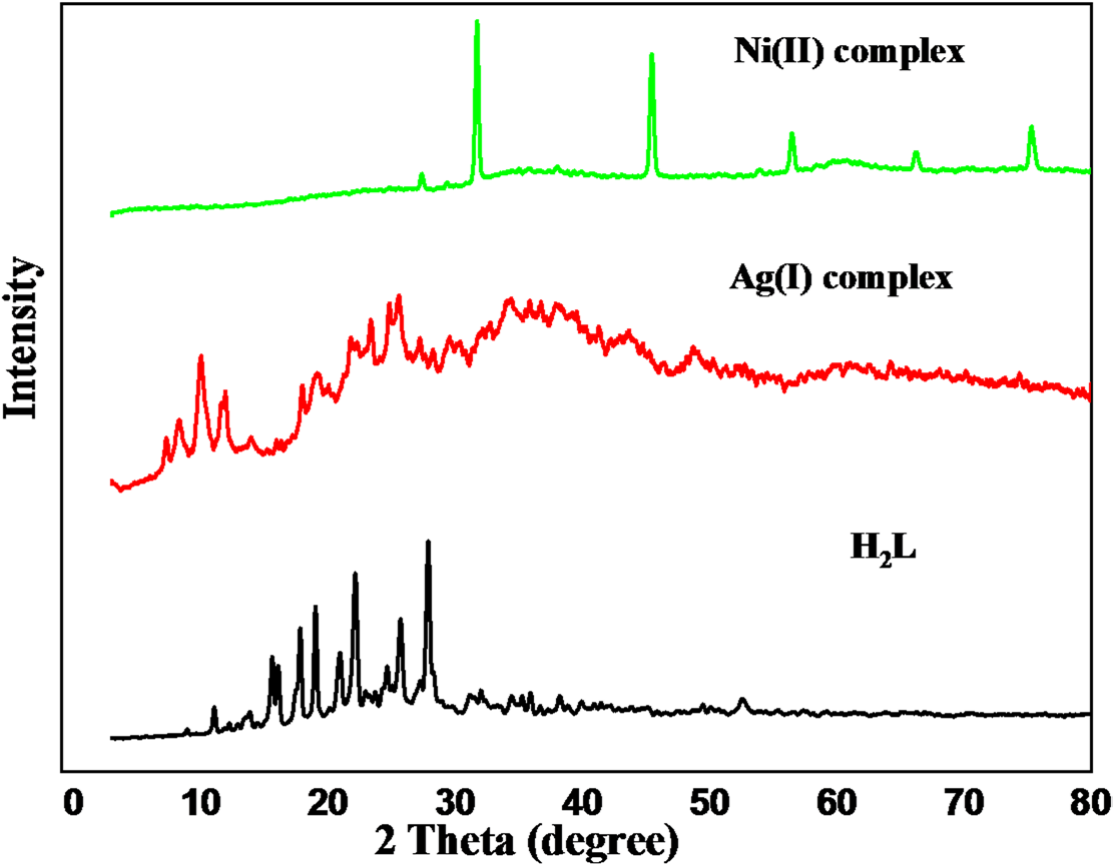
X-ray diffraction (XRD) patterns of H₂L ligand and its Ag(I) and Ni(II) complexes, showing their crystalline nature.

H_2_L and its complexes were subjected to X-ray diffraction at room temperature, with 2Ɵ values ranging from 10° to 80°, as shown in Fig. 7. In addition to the appearance of new peaks for 2Ɵ values, there are also discernible differences in the relative positions and intensity of some peaks. This alteration could be explained by the production of new phases as a result of metal complex formation and metal ion bonding^49^. Crystallite size (Cs), dislocation density (D), and microstrain (ɛ) of the synthesized compounds were computed using Debye-Scherrer Eq^50^.

$$\:\mathrm{C}\mathrm{s}=\:\:\frac{K{\uplambda\:}}{{\upbeta\:}\mathrm{c}\mathrm{o}\mathrm{s}{\uptheta\:}}\:\:\:\:\:\:\:\:\:\:\:\:\:\:\:\:\:\:\:\mathrm{D}=\frac{1}{{\mathrm{C}}_{s}^{2}}\:\:\:\:\:\:\:\:\:\:\:\:\:\:\:\:\:\:\:{\upvarepsilon\:}=\frac{{\upbeta\:}}{4\mathrm{t}\mathrm{a}\mathrm{n}{\uptheta\:}}\:\:$$

The X-ray beam’s wavelength is λ (1.5406 Å), its full width at half maximum (FWHM) is β (radians), its angle of diffraction is θ (radians), and the Scherrer constant is k = 0.9^50^. Cs measurements revealed nanostructures for produced compounds; the calculated values of Cs for the H_2_L, Ag(I), and Ni(II) complexes were 49.76, 85.06, and 58.74 nm, respectively. Dislocation density (D) values are 4.03, 1.38, and 2.89 × 10^− 4^ (nm^− 2^), and microstrain (ɛ) is 30.28, 19.86, and 22.61 × 10^− 4^ for H_2_L, Ag(I), and Ni(II), respectively. XRD analysis indicates that the Ag(I) complex exhibits broad peaks, signifying low crystallinity. While in Ni(II) coordination, sharper peaks emerge, indicating increased crystallinity. The Ni(II) complex displays even more intense and defined peaks, reflecting a higher degree of structural order. Thus, crystallinity increases from H₂L to the Ni(II) complex, suggesting Ni(II) induces the most significant structural ordering. The X-Pert High Score Plus program was used to determine the crystallographic parameters of H₂L and its metal complexes. The data collected are summarized in Tables 6, 7 and 8.

**Table 6:** Crystallography data of H_2_L.

System: Monoclinic a = 10.5300 Å b = 13.1930 Å c = 17.0464 Å α = 90° β = 108.02° γ = 90°			
N	2Ɵ	d Å	(hkl)
1	11.082	7.97788	−111
2	16.045	5.51956	102
3	17.695	5.00841	−202
4	18.938	4.68236	−212
5	22.058	4.02645	130
6	25.878	3.44021	−133
7	27.576	3.23202	041

**Table 7:** Crystallography data of Ag(I) complex.

System: Monoclinic a = 10.5650 Å b = 6.8920 Å c = 12.2560 Å α = 90° β = 95.57° γ = 90°			
N	2Ɵ	d Å	(hkl)
1	7.241	12.19807	001
2	8.402	10.51506	100
3	10.552	8.37685	−101
4	11.623	7.60740	101
5	23.368	3.80370	202
6	25.833	3.44600	020

**Table 8:** Crystallography data of Ni(II) complex.

System: Anorthic a = 9.1410 Å b = 14.0900 Å c = 14.1880 Å α = 94.82° β = 92.36° γ = 107.67°			
N	2Ɵ	d Å	(hkl)
1	27.253	3.26962	−232
2	31.448	2.84239	−141
3	45.46	2.63691	034
4	56.34	2.62907	105
5	75.16	2.61517	302

### Density functional theory (DFT) study

**Fig. 8:**
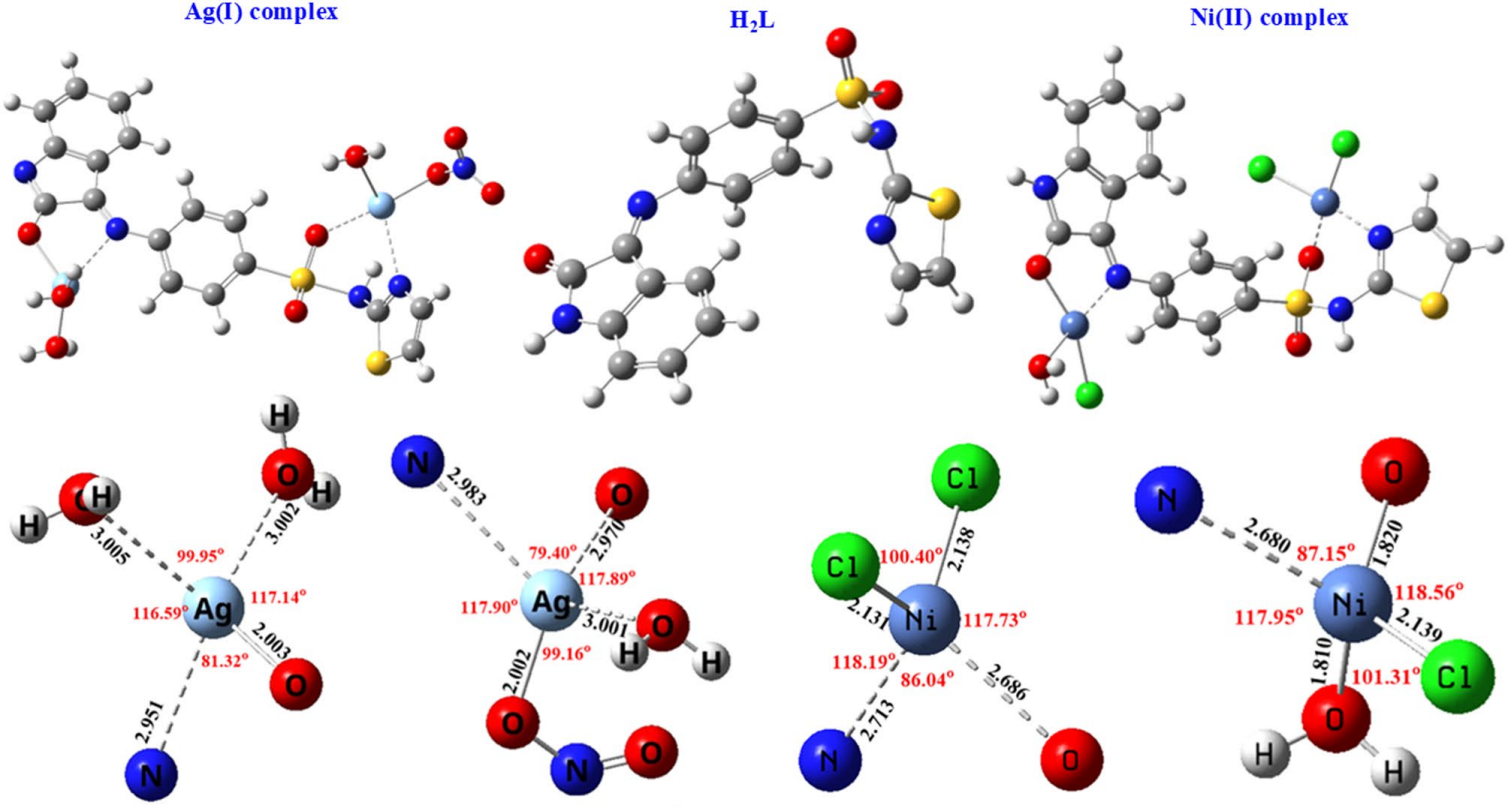
The optimized geometries of H₂L ligand and its Ag(I) and Ni(II) complexes, showing key bond lengths (Å) and bond angles (°).

**Fig. 9:**
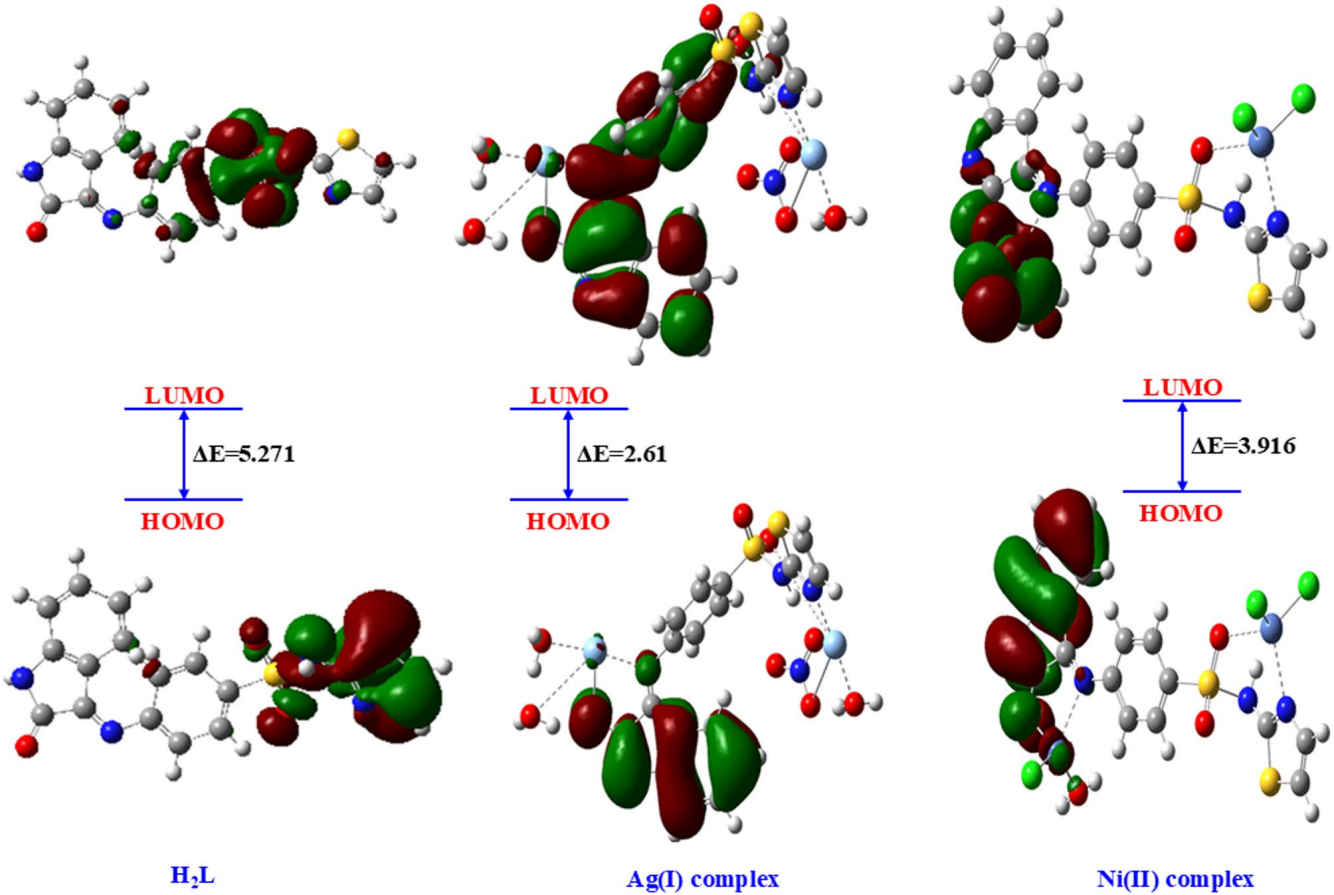
The molecular orbitals at the frontier for the H₂L ligand and its Ag(I) and Ni(II) complexes.

Density Functional Theory (DFT) has emerged as a crucial tool in quantum chemistry for precisely modeling molecular structures and properties. According to DFT calculations, the optimum geometry and boundary molecular orbitals of H_2_L and its complexes are depicted in Figs. 8 and 9. Also, bond lengths and angles of metal complexes are presented in Fig. 8. HOMO and LUMO, which function as electron donors and acceptors, respectively, are key parameters for understanding the electronic behavior of molecules^51,52^. Molecular chemical stability and reactivity are indicated by the energy gap, which is the difference between HOMO and LUMO. A larger energy gap generally corresponds to higher molecular stability, whereas a smaller gap indicates enhanced chemical reactivity^53,54^. The energy band gaps are 2.61 and 3.916 eV for the Ag(I) complex and Ni(II) complex, compared to 5.271 eV for H₂L, suggesting increased molecular reactivity for metal complexes compared to H_2_L^55^. Table 9 presents various chemical descriptor parameters. According to computational data, the metal complexes have better thermodynamic stability and improved electrical characteristics than the unchelated ligand, which supports their potential for increased chemical and biological activity.

**Table 9:** DFT-calculated global characteristics of chemical reactivity for H₂L ligand and its Ag(I) and Ni(II) complexes.

Molecular Characteristics (unit)	H_2_L	Ag(I) complex	Ni(II) complex
E total (a.u.)	−1886.663	−2696.635	−3691.0217
E_HOMO_ (eV)	−6.779	−5.032	−6.265
E_LUMO_ (eV)	−1.508	−2.422	−2.349
ΔE Energy gap (eV)	5.271	2.61	3.916
Ionization potential IP (eV)	6.779	5.032	6.265
Electron affinity EA (eV)	1.508	2.422	2.349
Absolute electronegativity χ (eV)	4.144	3.727	4.307
Absolute hardness η (eV)	2.635	1.305	1.958
Chemical potentials µ (eV)	−4.144	−3.727	−4.307
Global softness S (eV) ^−1^	1.318	0.653	0.979
Global electrophilicity ω (eV)	3.258	5.321	4.737
Additional electronic charge N max	1.572	2.856	2.2
Dipole moment (Debye)	5.615	8.089	12.66

### Mulliken atomic charge

The calculated Mulliken charges are visualized in Fig. 10**(a-c)**. More negatively charged atoms behave as nucleophilic sites, contributing their extra electrons to a metal’s vacant orbital^56,57^. O_9_, N_14_, N_16_, and O_22_ are the atoms in the unbound H₂L ligand that are most negatively charged. The Mulliken charge distribution analysis of Ag(I) and Ni(II) complexes indicates that the nitrogen atom in the azomethine group, the oxygen atom in the isatin moiety, the nitrogen atom of the thiazole group, and the oxygen atom of the sulfonamide exhibit lower negative charges compared to the H_2_L ligand. Electron transfer from the free ligand to the metal atom reduces the negative charges at azomethine-N, isatin moiety-O, thiazole-N, and sulfonamide-O atoms in the complexes relative to the H_2_L ligand.

**Fig. 10:**
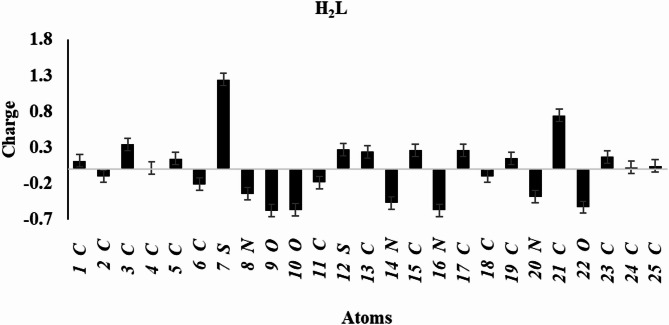
**(a)**: Mulliken atomic charges of the H_2_L ligand.

**Fig. 10:**
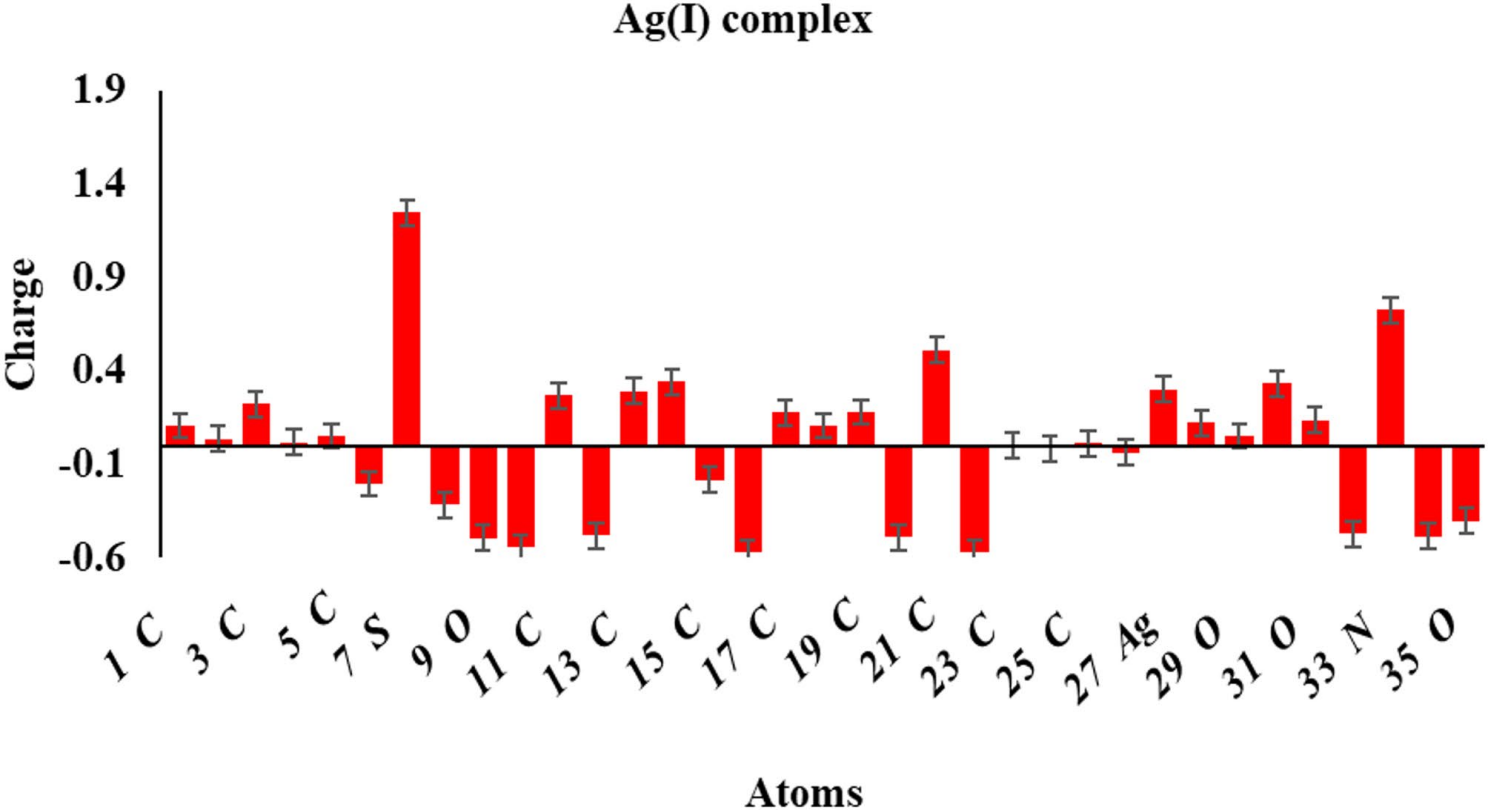
(b): Mulliken atomic charges of the Ag(I) complex.

**Fig. 10:**
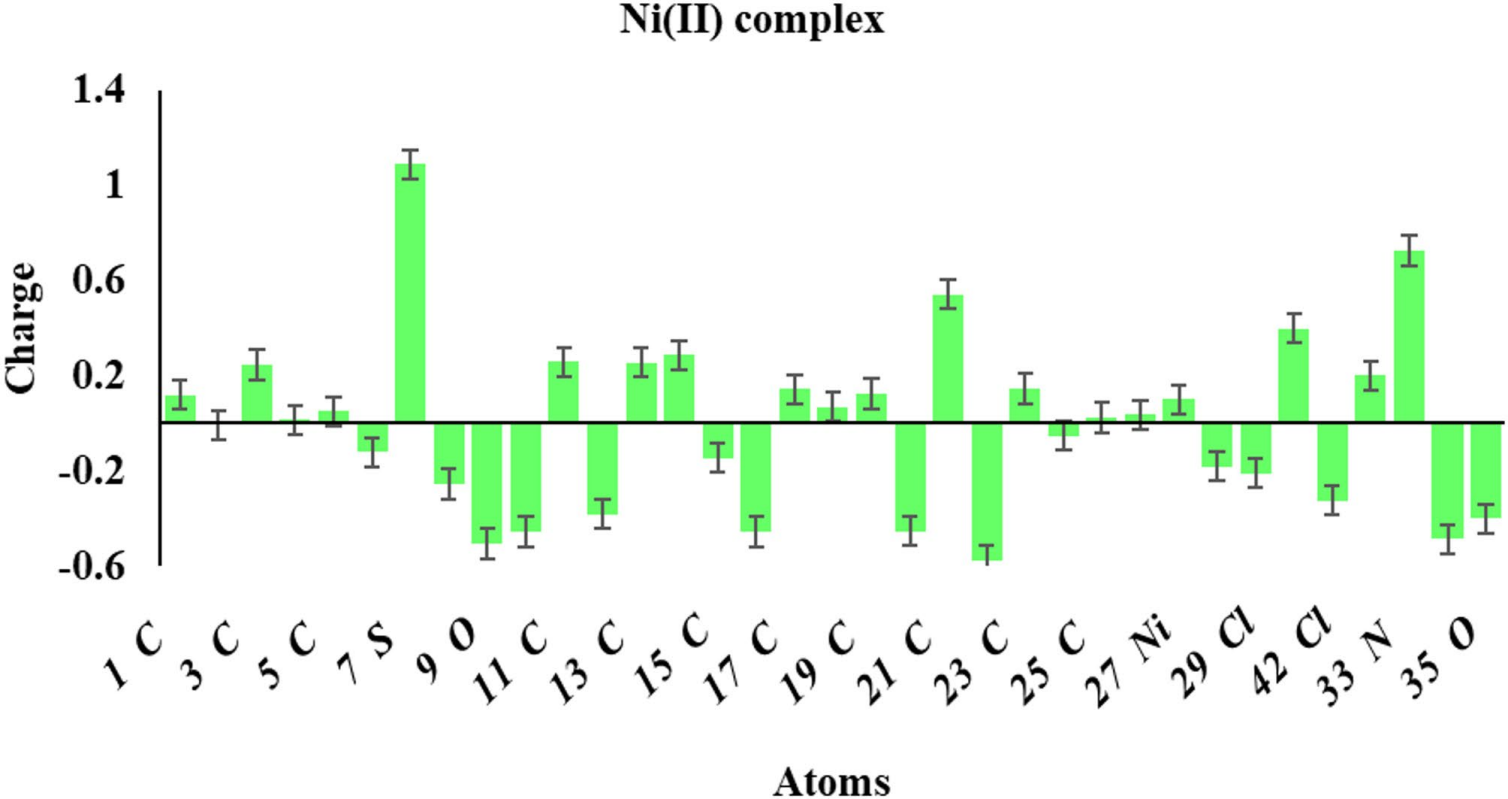
(c): Mulliken atomic charges of the Ni(II) complex.

### Molecular electrostatic potential (MEP/ESP)

**Fig. 11:**
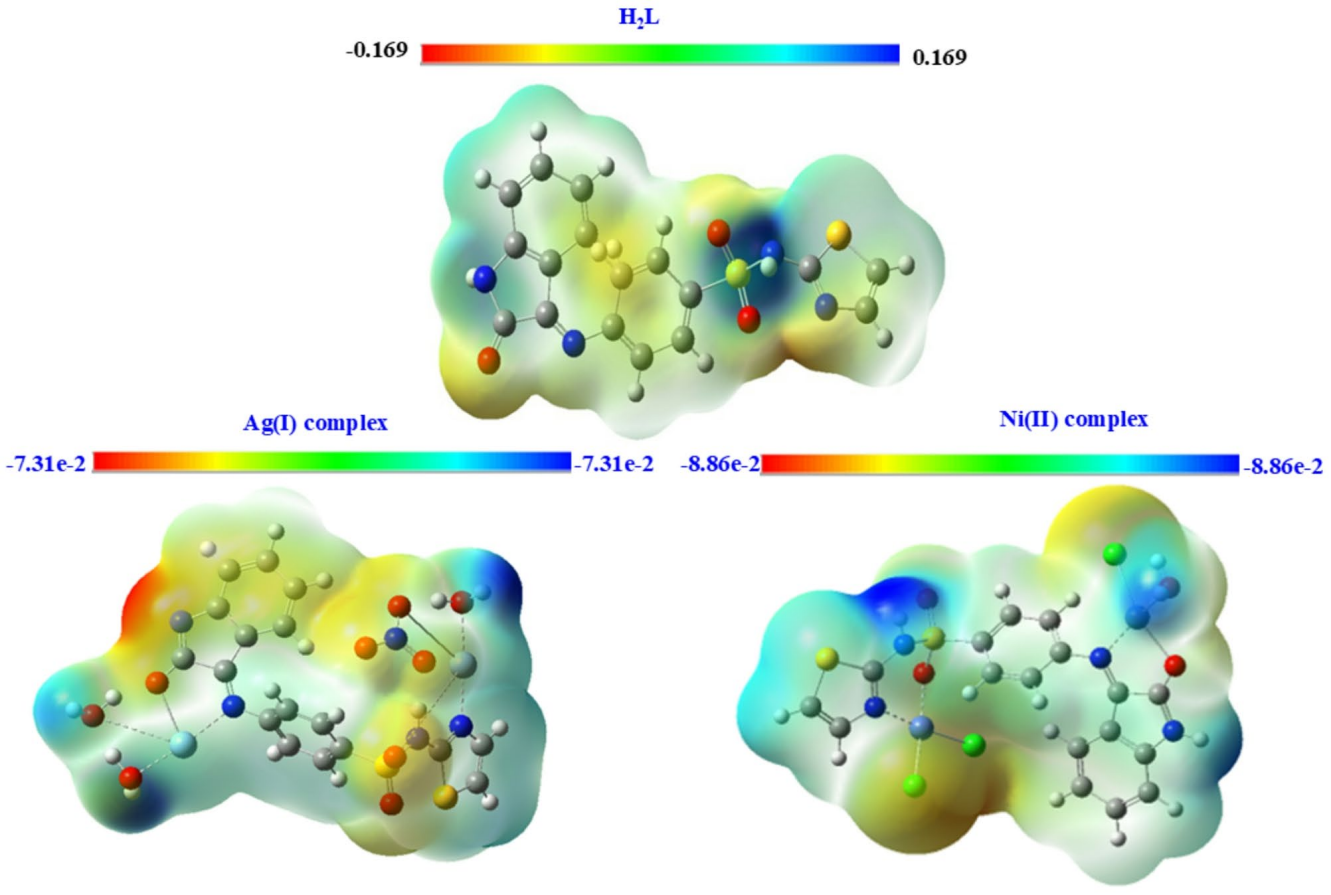
The molecular electrostatic potential for the H₂L ligand and its Ag(I) and Ni(II) complexes.

To predict charge distribution and locate reactive sites for electrophilic and nucleophilic attacks, the molecular electrostatic potential (MEP) offers crucial information on the electron density distribution inside molecular systems. The potential follows a blue-green-red pattern, where blue implies the strongest attraction and red signifies the strongest repulsion, as shown in Fig. 11. By examining MEP visualizations, researchers can discover possible electrophilic and nucleophilic interaction sites and provide important insights into chemical reactivity and site selection^58,59^.

### MB dye degradation by photocatalysis

#### Determining the point of zero charge

**Fig. 12:**
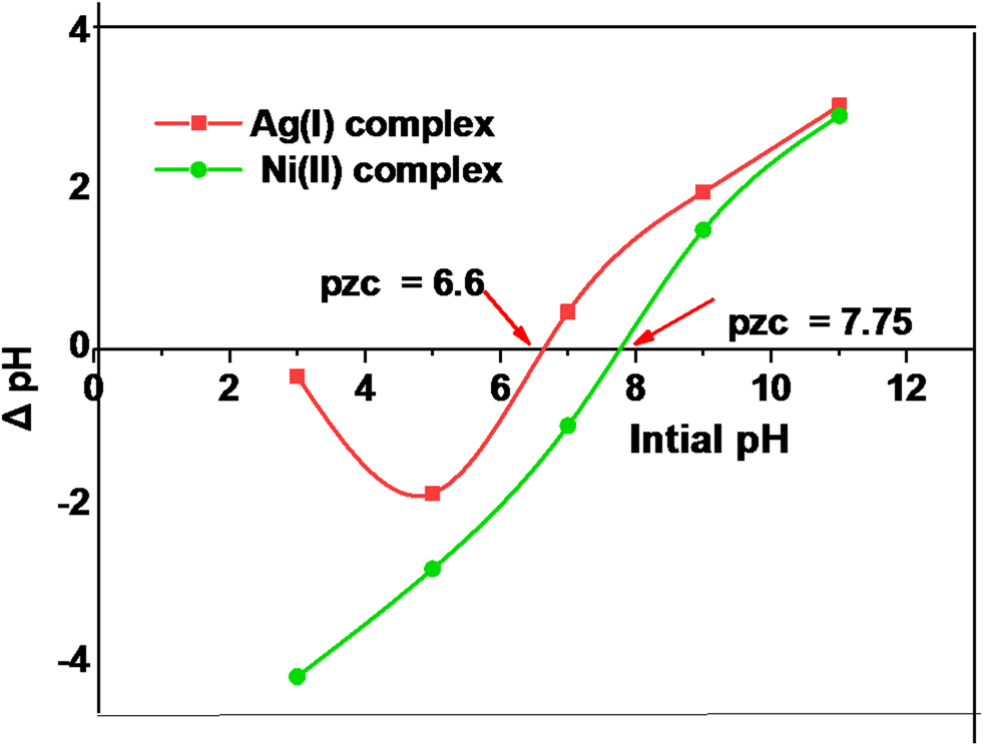
Point of zero charge (PZC) determination for Ag(I) and Ni(II) complexes.

The point of zero charge (pHpzc) of Ag(I) and Ni(II) complexes was ascertained in accordance with the technique to clarify the impact of pH^6,60^, where the pHpzc for Ag(I) and Ni(II) complexes was determined to be ~ 6.6 and 7.75, respectively, **as in** Fig. 12. At pH below pHpzc, the photocatalyst surface has a positive charge, and at pH above pHpzc, the surface has a negative charge. In addition, the point zero charge measurement implies that the persistent negative charge on the surface of the Ag(I) and Ni(II) complexes may be responsible for the enhanced photocatalytic degradation of the cationic dye MB. Because of its negative charge, the cationic MB dye can adsorb more effectively. At solution pH values below the pHpzc of both complexes, the positive charge density of the complexes and MB molecules resulted in significant electrostatic repulsion, leading to reduced MB adsorption on the surface of the complexes. Consequently, the interaction between Ag(I) and Ni(II) complexes and MB dye, as well as the photocatalytic degradation of MB, was affected by the variation in solution pH.

### Influence of catalyst dosage

**Fig. 13:**
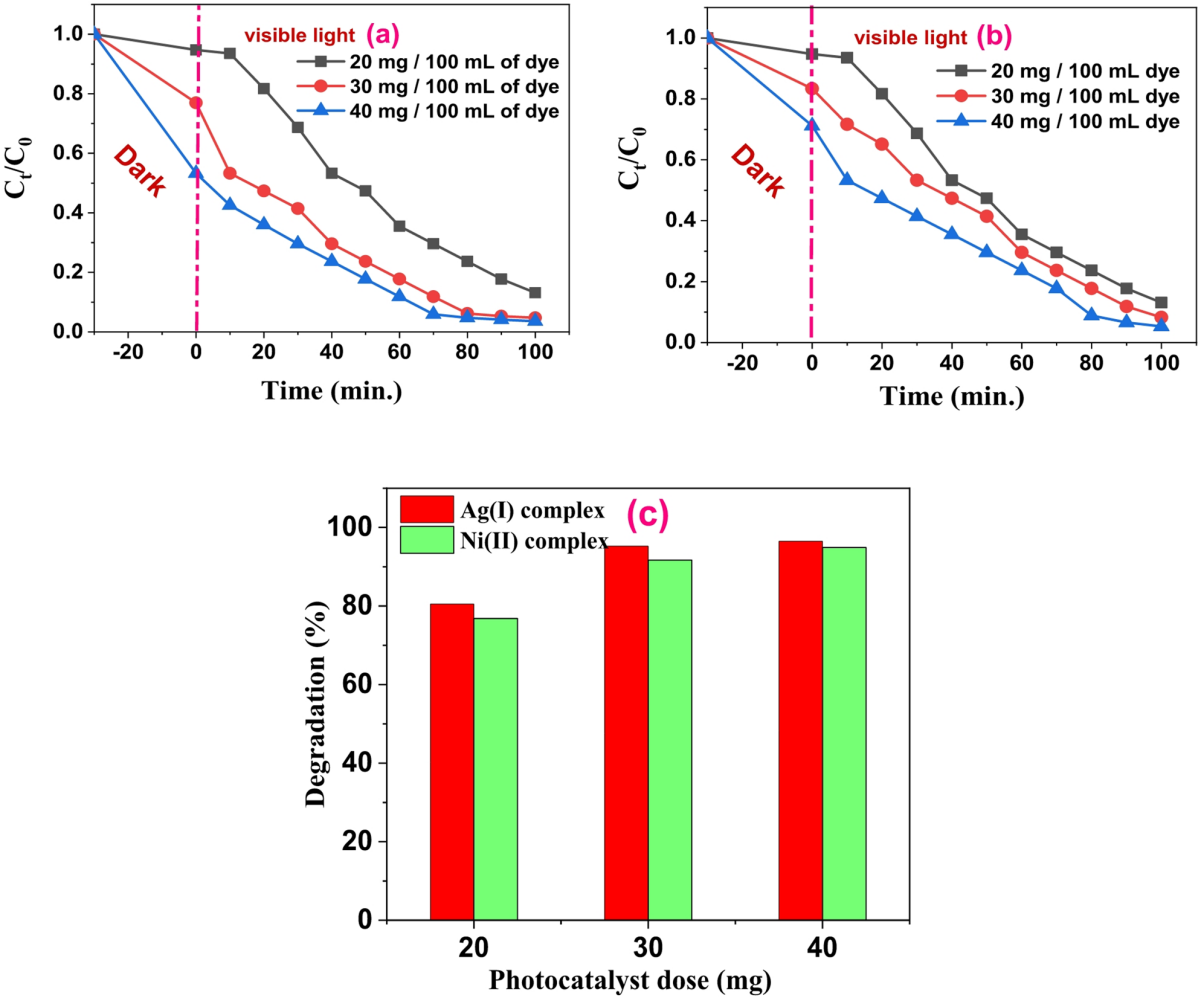
**(a)** and **(b)** Effect of catalyst dose of Ag(I) and Ni(II) complexes on the degradation of Mb dye at 10 ppm and pH 11 solution under visible light irradiation. **(c)** Photo degradation % graph of MB dye reaction over Ag and Ni complexes at different dosages.

The catalyst’s dose has a significant impact on photocatalytic processes. To investigate the impact of the catalyst dosage on the photocatalytic efficacy, 100 mL of 10 ppm of MB dye solution at pH 11 was used with three different catalyst doses (20, 30, and 40 mg) of Ag(I) and Ni(II) complexes. Increasing the catalyst’s quantity makes more active sites available to interact with contaminants, improving the effectiveness of photocatalytic degradation. Conversely, if fewer catalysts were used, fewer photons would be absorbed, which would cause deterioration to proceed more slowly. As illustrated in Figs. 13a **and b**, the efficiency of Ag(I) and Ni(II) complexes in eliminating MB rose with increasing dosages of 20, 30, and 40 mg/100 mL dye solution after 100 min of exposure to visible light. As seen in Fig. 13c, increasing the dosage gradually from 30 mg/100 mL dye to 40 mg/100 mL dye only increased the removal efficiency by 3.19% for the Ni(II) complex and 1.3% for the Ag(I) complex. This could be because an excessive amount of photocatalyst blocked light penetration. Additionally, agglomeration may also take place. Higher concentrations of the catalyst decrease surface area and slow down catalytic efficiency^61^. Consequently, it was determined that 30 mg/100 mL dye was the ideal dosage for this study, taking into account both cost and effectiveness.

### Effect of dye concentration

**Fig. 14:**
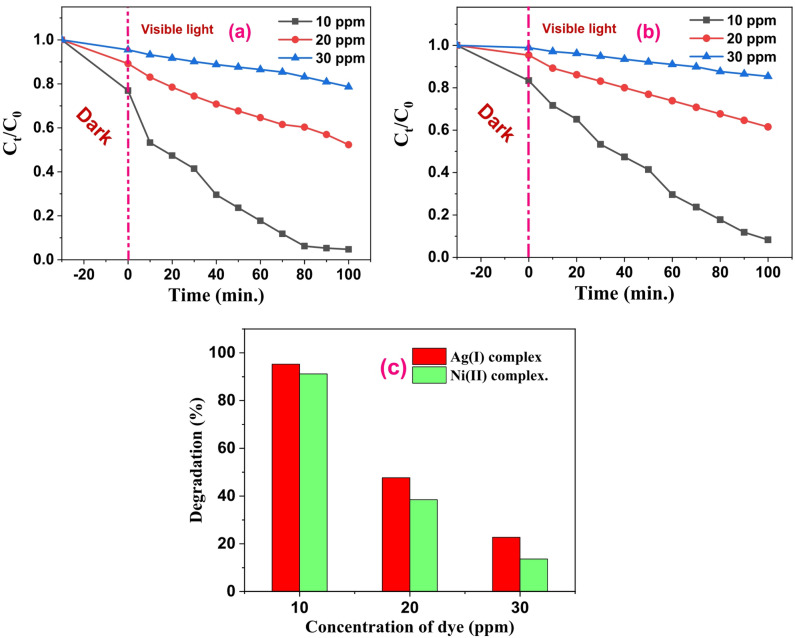
**(a)** and **(b)** Effect of dye concentrations of MB using Ag(I) and Ni(II) complexes as photocatalysts at 30 mg of photocatalyst/100 mL of dye solution and pH 11 solution under visible light irradiation, respectively. **(c)** Photodegradation % graph of MB dye reaction over Ag and Ni complexes at different dye concentrations.

To investigate the impact of the initial dye concentration on the photocatalytic efficacy, a fixed quantity (30 mg) of the catalyst was applied to three different starting dye concentrations (10, 20, and 30 ppm) of the MB dye solution. As seen in Figs. 14a **and b**, the dye solution’s pH was kept at 11 for the experiment. The degradation efficiency dropped from 95.3% to 22.72% for the Ag(I) complex and from 91.7% to 13.77% for the Ni(II) complex after increasing the dye solution from 10 to 30 ppm. Figure 14c. One explanation is that as more dye molecules are adsorbed onto the surface of the Ag(I) and Ni(II) complex nanoparticles, the nanoparticles’ active sites are completely saturated. The quantity of superoxide and hydroxyl radicals that can be adsorbed onto the catalyst is reduced because of this saturation. Additionally, as the dye solution becomes more vividly colored, the amount of light that can flow through it is reduced. As a result, the degradation efficiency decreases because fewer photons can interact with the dye molecules at the catalyst’s surface^62^. However, many of the complex nanoparticles’ active sites remain empty at low dye concentrations. The interaction between the dye and photons is facilitated as a result. Accordingly, deterioration efficiency increases as a result.

### Impact of the solution’s pH

**Fig. 15:**
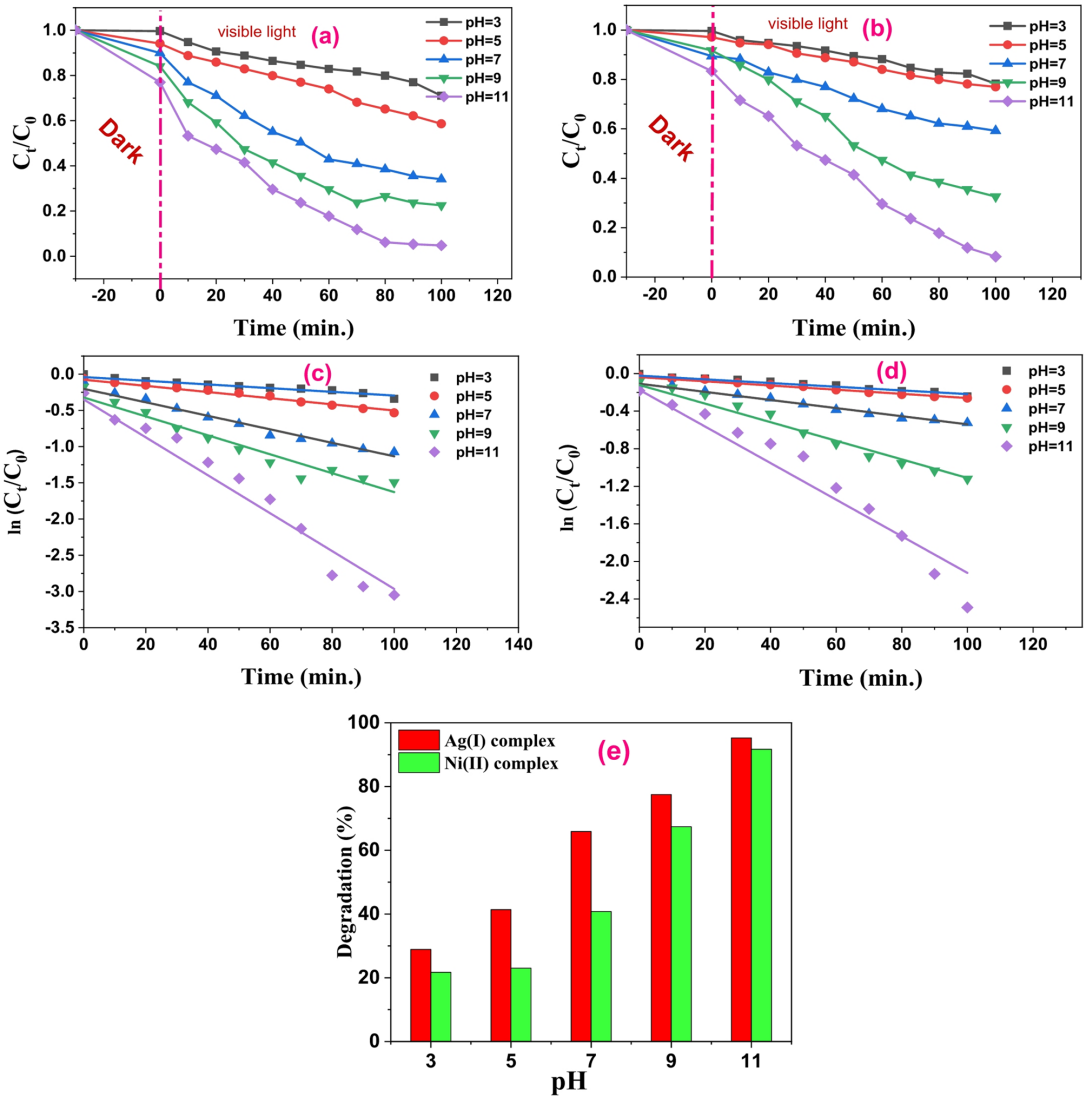
**(a)** and **(b)** Effect of pH value on photodegradation of MB using Ag(I) and Ni(II) complexes in 10 ppm dye concentration with 30 mg of photocatalyst/100 mL of dye solution under visible light irradiation, respectively. **(c)** and **(d)** Pseudo-first-order kinetics at different pH values for (a) Ag(**I**) complex and (d) Ni**(II)** complex. **(e)** Photodegradation % graph of MB dye reaction over Ag and Ni complexes at different pH.

A solution’s pH is essential for determining a catalyst’s photocatalytic efficiency since it provides information on the catalyst’s surface charge properties^63,64^. As a result, the MB dye solution’s degradation under various pH levels (3, 5, 7, 9, and 11) was investigated. The dye solution was adjusted by adding HCl or NaOH to get these different pH levels. Methylene Blue (MB) photodegradation at pH values of 3, 5, 7, 9, and 11 is shown in Figs. 15a, b for Ag(I) and Ni(II) complexes, respectively. As pH rises from 3 to 11, degradation efficiency increases. In acidic conditions (pH < 7), the removal effectiveness is relatively lower because complex compounds dissolve, and due to strong electrostatic repulsion caused by the high positive charge densities of the complex and MB, the adsorption of MB onto the two complex surfaces was reduced when the pH of the solution decreased below pH_pzc_. The results in Fig. 15**(e)** show that the highest degradation percentages were observed for the Ag(I) and Ni(II) complexes in an alkaline medium (pH = 11), at roughly 95.3% and 91.7%, respectively. Since the surface of the alkaline environment produces negatively charged particles, MB, a cationic dye, can complete the degradation process more quickly^65,66^. Efficient degradation primarily occurs at elevated pH levels, attributed to the cationic nature of the MB dye, which facilitates its attraction to the negatively charged catalyst surface^67^.

#### Kinetics of degradation of dye

The collected photodegradation data is fitted to first-order kinetics using Eq. (2) to ascertain the degradation rate:

$$\:\mathrm{ln}\frac{{C}_{t}}{{C}_{0}}\:={k}_{app}\:t\:\:\:\:\:\:\:\:\:\:\:\:\:\:\:\:\:\:\:\:\:\:\:\:\:\:\:\:\:\:\left(2\right)\:\:\:\:\:\:$$

where t is the irradiation time, *k*_*app*_ is the rate constant, and C_o_ and C_t_ are the starting and ending concentrations of MB dye during periodic time interruptions^68,69^. The slope of the linear plot of ln(C_t_/C_o_) against time (t) is used to calculate the value of *k*_*app*_. The plots’ straight line verified that, for all examined photocatalysts, the degradation of MB employing Ag(I) and Ni(II) complexes as photocatalysts followed pseudo-first-order kinetics at various pH levels, and hence both correlation coefficient R² values > 0.9 for the two complexes as seen in Figs. 15c **and d.** Table 10 lists the correlation coefficient (R²) and apparent rate constant for each photocatalyst when exposed to visible light. The Ag(I) and Ni(II) complexes completed the reaction in approximately 100 min. and have a value of k equal to 26.16 × 10^− 3^ and 19.49 × 10^− 3^ min.^−1^, respectively, at a pH of 11, which is very high compared to the acidic value. This result indicates that the decolorization of MB dye was larger using the Ag(I) complex than the Ni(II) complex.

**Table 10:** Rate constants of pseudo-first order with their corresponding R^2^ values for synthetic photocatalysts.

pH	Ag(I) complex		Ni(II) complex	
	-k_app_×10^− 3^	*R* ^2^	-k_app_×10^− 3^	*R* ^2^
3	2.55	0.9498	19.7	0.9519
5	4.26	0.97768	22.3	0.97844
7	9.32	0.97877	4.33	0.99025
9	13.07	0.9603	9.9	0.96081
11	26.16	0.95318	19.49	0.9247

#### Recyclable nature of the photocatalyst

**Fig. 16:**
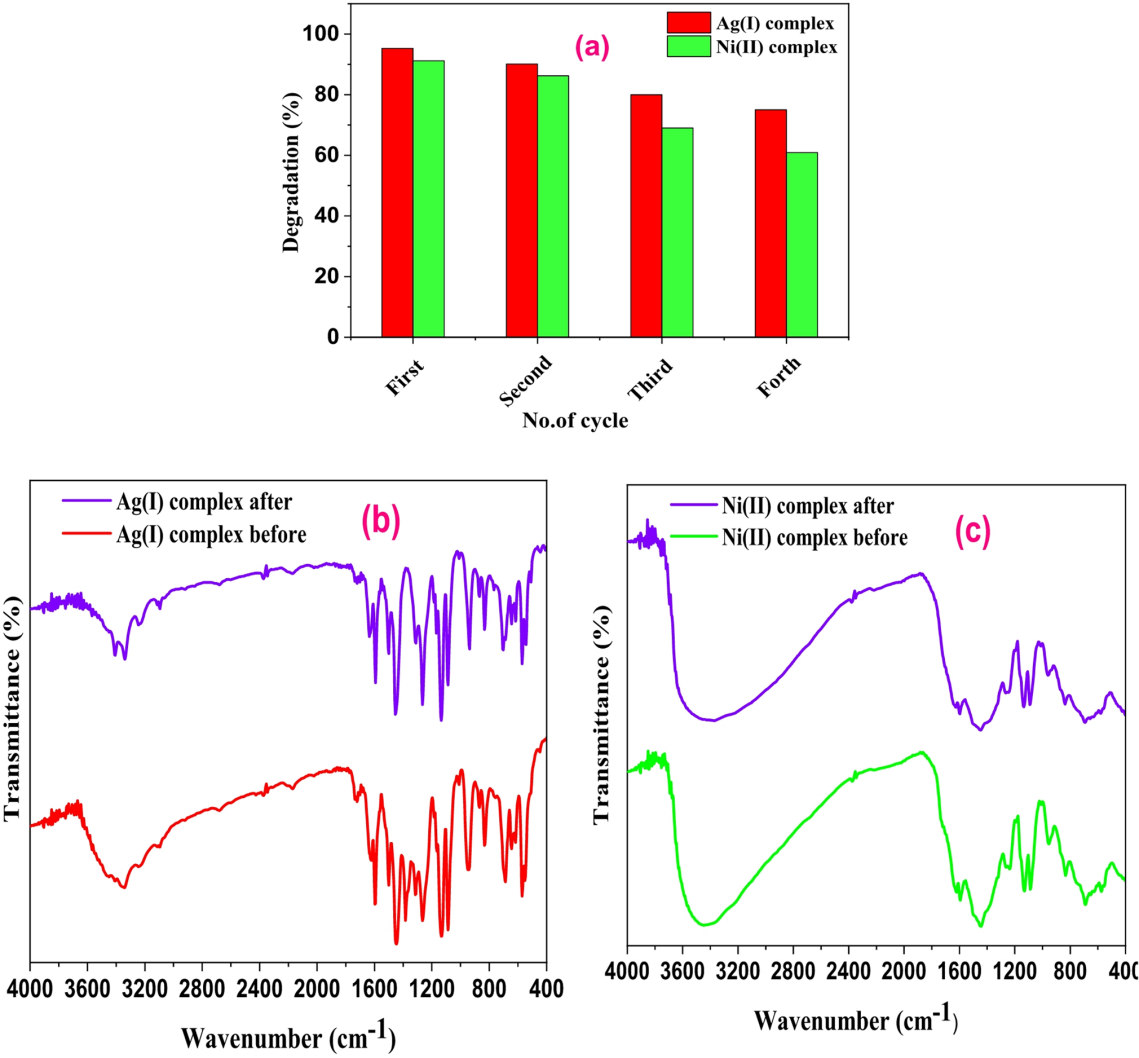
**(a)** Reusability of Ag(I) and Ni(II) complexes for four cycles during the visible light irradiation of MB dye. **(b)** and **(c)** FTIR of Ag(I) and, Ni(II) complexes before and after photocatalysis process.

The dispersed photocatalyst was separated by centrifugation after the photoreactions were finished, and it was found that the photocatalyst remained nearly the same color as before the photocatalysis process. This suggests that organic dye toxins were not introduced into the catalyst surface. After which, the pollutants were all mineralized. Four different batches of photoreactions were conducted using the photocatalysts. During subsequent runs, activity decreased somewhat, as seen in Fig. 16a. Because some catalyst is lost during the recovery process, causing a gradual decline in activity^70^. Ag(I) and Ni(II) complexes have excellent reusability performance and longevity under visible light irradiation, making them a promising candidate in practical applications, including wastewater treatment. The photocatalytic performance of the synthesized complexes was observed to show no discernible decay with a prolonged number of cycles up to 4, showing that they have outstanding cyclic stability. To assess the stability of the two complexes, comparisons were made between the FTIR results of the freshly synthesized complexes and those of the recovered ones Fig. 16b **and c**. There were no discernible differences between the recovered and newly made catalysts, according to the results. Consequently, it can be said that Ag(I) and Ni(II) complexes show exceptional stability and can act as a photocatalyst to degrade MB.

#### Proposed mechanism of dye degradation using Ag(I) and Ni(II) complexes/DFT insights

**Scheme 2:**
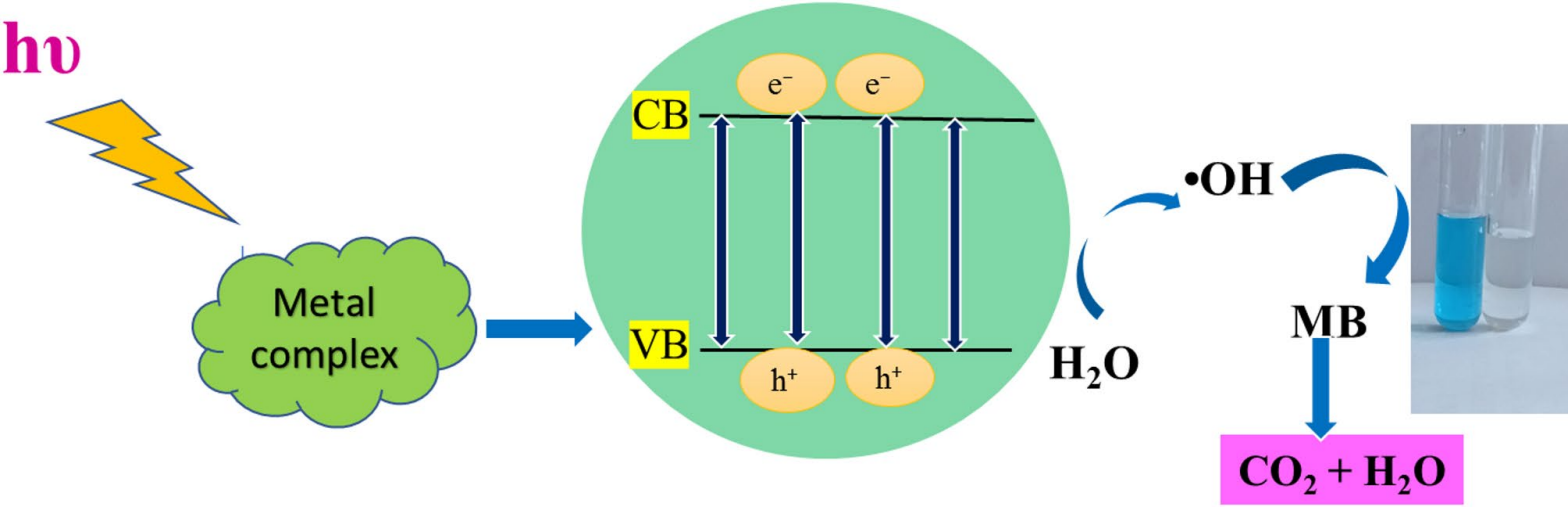
The schematic representation of the proposed photocatalytic degradation mechanism for the degradation of Methylene Blue (MB) dye using the synthesized metal complex.

The mechanism of photocatalytic degradation of dyes in the presence of Ag(I) or Ni(II) complex has been investigated. Under visible light irradiation, electrons would transfer from the valence band (VB) to the conduction band (CB) of the complex, resulting in the generation of holes (h^+^) in the VB of the complex. To counteract this imbalance, the O_2_ molecule might trap one electron from CB to form ^**•**^O_2_^‒^ species, which is further reduced to form ^•^ OH active species. Also, holes present in VB might capture one electron from a water molecule to generate ^**•**^OH active species. The ^•^ OH generated on visible light irradiation effectively breaks down MB molecules to form the final oxidation products, acting as a powerful oxidizing agent. The interaction of these hydroxyl radicals with MB molecules results in the collapse of the MB structure. There are many sites in the structure of dye molecules that are repeatedly attacked by the ^•^ OH radicals that are generated, where they are converted to less harmful intermediate products. The mechanics of the photocatalytic process can be explained using Eqs. (3–7) and Scheme 2^42,67,71^:

2 $${\mathbf{metal}}{\text{ }}{\mathbf{complex}} + {\text{ }}{\mathbf{h\upsilon }} \to {\text{ }}{\mathbf{metal}}{\text{ }}{\mathbf{complex}}{\text{ }}\left( {{{\mathbf{e}}^ - } + {\text{ }}{{\mathbf{h}}^ + }} \right)$$

3 $${\mathbf{e}} - {\text{ }} + {{\mathbf{O}}_{\mathbf{2}}}{ \to ^ \bullet }{{\mathbf{O}}_{\mathbf{2}}}^ -$$

4 $${{\mathbf{h}}^ + } + {\text{ }}{{\mathbf{H}}_{\mathbf{2}}}{\mathbf{O}}{\text{ }}{ \to ^ \bullet }{\mathbf{OH}}{\text{ }} + {\text{ }}{{\mathbf{H}}^ + }$$

5 $$^ \bullet {{\mathbf{O}}_{\mathbf{2}}}^ - + {\text{ }}{{\mathbf{H}}_{\mathbf{2}}}{\mathbf{O}}{\text{ }} \to {\text{ }}{\mathbf{H}}{{\mathbf{O}}_{\mathbf{2}}}^ \bullet$$

6 $$^ \bullet {{\mathbf{O}}_{\mathbf{2}}}^ - + {\text{ }}{{\mathbf{H}}_{\mathbf{2}}}{\mathbf{O}}{\text{ }} \to {\text{ }}{\mathbf{H}}{{\mathbf{O}}_{\mathbf{2}}}^ \bullet$$

7 $$^ \bullet {\mathbf{OH}}{\text{ }} + {\text{ }}{\mathbf{MB}}{\text{ }} \to {\text{ }}{\mathbf{Degradation}}{\text{ }}{\mathbf{products}}$$

### DFT insights

The photocatalytic performance of the Ag(I) and Ni(II) complexes can be rationalized based on their electronic properties obtained from DFT calculations. The calculated HOMO–LUMO energy gaps are 2.61 eV for Ag(I) and 3.916 eV for Ni(II), significantly lower than 5.271 eV for the free ligand (H₂L). The smaller band gaps facilitate efficient visible-light absorption and promote electron–hole pair generation, which is essential for the formation of reactive species responsible for dye degradation. Additionally, the Mulliken charge analysis reveals that electron-rich regions on the complexes, particularly at the azomethine nitrogen, isatin oxygen, thiazole nitrogen, and sulfonamide oxygen, favor strong electrostatic interactions with the cationic (MB) molecules. This enhances adsorption onto the catalyst surface and improves subsequent photodegradation. The combination of a lower band gap and favorable charge distribution explains why the Ag(I) complex exhibits slightly higher photocatalytic efficiency (95.3%) than the Ni(II) complex 91.7% under optimized conditions (pH 11, 30 mg catalyst/100 mL, 10 ppm MB). Thus, both electronic and surface charge properties of these complexes play a crucial role in their superior photocatalytic performance.

### Comparison with previous studies

The unique contribution of this work is highlighted by comparing performance under normalized experimental conditions, as summarized in Table 11. While many previously reported Schiff base metal complexes achieve high efficiencies under intensified parameters such as high catalyst loadings, UV or high-power lamps, or the addition of external oxidants like H₂O₂, their intrinsic activity is often masked by these favorable conditions. In contrast, the nanoscale Ag(I) and Ni(II) complexes presented here demonstrate superior intrinsic photocatalytic efficiency, primarily driven by structural novelty. The sonochemical synthesis method produces nanoscale complexes with optimized electronic structures and high surface area, distinguishing them from conventionally prepared systems. Importantly, our complexes achieve degradation efficiencies of 95.3% and 91.7% under visible light without any external oxidant (H₂O₂-free), using moderate catalyst dosages and shorter irradiation times. These results confirm that the observed high performance arises from the optimized nanoscale morphology and intrinsic electronic properties induced by the sonochemical route, rather than from externally assisted reaction conditions. This distinctive synthetic strategy, combined with the resulting high efficiency under mild conditions, establishes the clear novelty and significance of the present study.

### Limitations of the study

The present study is limited to the photocatalytic degradation of methylene blue under laboratory conditions. The performance of the catalysts toward other pollutants and in real wastewater systems was not investigated. Moreover, recyclability was evaluated over a limited number of cycles. These aspects will be addressed in future studies.

**Table 11:** Comparison of reported schiff base metal complexes for photocatalytic degradation of methylene blue (MB) with the present work.

Metal complex	Synthesis method	Experimental conditions	Degradation (%)	Ref.
Nanoscale Ag(I) and Ni(II) complexes of isatin–sulfathiazole ligand	Sonochemical method	MB = 10 ppm; catalyst = 30 mg; pH = 11; visible light (60 W tungsten lamp); H₂O₂-free system; irradiation time = 100 min	95.3, 91.7	Current work
[Co (L_1_)_2_], [Cu(L_1_)_2_], [Co(L_2_)_2_], [Cu(L_2_)_2_], [Co(L_3_)_2_], [Cu(L_3_)_2_] complexes of azo-aldehydes derivative Schiff base ligands	conventional reflux method	MB = 15 ppm; catalyst = 10 mg, 200 W LED visible light irradiation; irradiation time = 105 min	61, 92, 79, 93, 57, 93	^72^
Mixed-ligand Cu(II) Schiff base complex	conventional reflux method	MB = 40 mg L⁻¹; catalyst = 0.5 g; pH = 10; T = 333 K; UV irradiation; irradiation time = 40 min	70.71	^73^
[Ni(MPAFA)₃](BF₄)₂ and [Co(MPAFA)₃](BF₄)₂	conventional reflux method	MB = 10 mg L⁻¹; catalyst dosage = 0.015 mmol; UV and visible light; irradiation time = 140 min	90.5, 89.6	^74^
Zn(II), Co(II), and Mn(II) complexes of 9-anthracenyl-4′-benzoate	conventional reflux method	MB = 5 mg L⁻¹; catalyst = 10 mg; visible light (500 W halogen lamp); irradiation time = 120 min	78.64, 83.33, 91.5	^75^
Zn(II) and Cu(II) Schiff base complexes	conventional reflux method	MB = 10 mg L⁻¹; catalyst = 50 mg; visible light (200 W Hg lamp); irradiation time = 120 min; 1 mL of 30% H₂O₂ added	96.0, 91.5	^76^
Pyrimidine-based Co(II), Ni(II), and Cu(II) Schiff base complexes	conventional reflux method	MB = 7 mg L⁻¹; catalyst dosage = 0.02 mmol; UV irradiation; irradiation time = 120 min	86.7, 84.5, 81.9	^77^
[Cd(H₄L¹)(N₃)₂]ₙ and [Cd₂(L²)(N₃)₂] **complexes**	conventional reflux method	MB = 10 µM; catalyst concentration = 1 g L⁻¹; UV irradiation; irradiation time = 180 min	37.64, 32.60	^78^
Co(II), Ni(II) complexes of pyrazolyl Schiff base ligand	conventional reflux method	MB = 10 mg L⁻¹; catalyst = 0.02 mmol; UV irradiation, irradiation time = 105 min	91.3, 93.8	^79^

## Conclusions

In this study, Ag(I) and Ni(II) Schiff base complexes derived from isatin and sulfathiazole were successfully synthesized via a green approach. The structures of the complexes were confirmed using elemental analysis, molar conductivities, magnetic moment, FT-IR spectroscopy, ¹H NMR spectroscopy, UV-visible spectroscopy, XRD, TGA, and mass spectroscopy. Thermogravimetric and DFT analyses demonstrated good thermal stability and favorable electronic properties, with reduced HOMO–LUMO energy gaps indicating enhanced electronic conductivity. The semiconductor behavior of the ligand and its Ag(I) and Ni(II) complexes was confirmed through optical band gap analysis. The photocatalytic performance of the complexes was evaluated through the degradation of methylene blue (MB) dye under visible light. Both complexes exhibited high photocatalytic efficiency, achieving optimal degradation under alkaline conditions (pH 11) using 30 mg of catalyst per 100 mL of a 10 ppm MB solution. Notably, the Ag(I) and Ni(II) complexes achieved degradation efficiencies of 95.3% and 91.7%, respectively, within 100 min. Kinetic analysis confirmed that the degradation process follows pseudo-first-order behavior. Furthermore, the complexes showed excellent reusability, maintaining high catalytic activity over four successive cycles. These findings highlight the potential of the synthesized complexes as efficient and reusable visible-light photocatalysts for wastewater treatment applications.

## Supplementary Information

Below is the link to the electronic supplementary material.

Supplementary Material 1

## Data Availability

The data supporting the findings of this study are available from the corresponding author, Aml M. Saleh, upon reasonable request (email: aml.mahmoud@azhar.edu.eg).
