# Supplementary material for: “Sonochemically synthesized Ag(I) and Ni(II) schiff base complexes as efficient visible-light photocatalysts for dye degradation with DFT insights.”
Source: Sci Rep. 2026 Feb 18;16:7181. doi: 10.1038/s41598-026-37498-8 (PMC12921343; doi:10.1038/s41598-026-37498-8)
Supplement: Supplementary file 1 — Supplementary Material 1 [file 41598_2026_37498_MOESM1_ESM.docx]

**"Sonochemically Synthesized Ag(I) and Ni(II) Schiff Base Complexes: DFT Insights and Efficient Visible-Light Photocatalysis for Dye Degradation"**

**Aml M. Saleh*, Amal G. Mahdy, Asmaa A. Hamed**

Chemistry Department, Faculty of Science (Girls), Al-Azhar University, Nasr City, Cairo, Egypt.

S1: Mass fragmentation of H_2_L

S2: Mass fragmentation of Ag(I) complex

S3: Mass fragmentation of Ni(II) complex
